# Analysis of Race and Ethnicity, Socioeconomic Factors, and Tooth Decay Among US Children

**DOI:** 10.1001/jamanetworkopen.2023.18425

**Published:** 2023-06-15

**Authors:** Sung Eun Choi, Joel White, Elizabeth Mertz, Sharon-Lise Normand

**Affiliations:** 1 Department of Oral Health Policy and Epidemiology, Harvard School of Dental Medicine, Boston, Massachusetts; 2 Department of Preventive and Restorative Dental Sciences, School of Dentistry, University of California, San Francisco; 3 Department of Health Care Policy, Harvard Medical School, Boston, Massachusetts; 4 Department of Biostatistics, Harvard T.H. Chan School of Public Health, Boston, Massachusetts

## Abstract

**Importance:**

While large oral health disparities remain by race and ethnicity among children, the associations of race, ethnicity, and mediating factors with oral health outcomes are poorly characterized. Identifying the pathways that explain these disparities would be critical to inform policies to effectively reduce them.

**Objective:**

To measure racial and ethnic disparities in the risk of developing tooth decay and quantify relative contributions of factors mediating the observed disparities among US children.

**Design, Setting, and Participants:**

This retrospective cohort study used electronic health records of US children from 2014 to 2020 to measure racial and ethnic disparities in the risk of tooth decay. Elastic net regularization was used to select variables to be included in the model among medical conditions, dental procedure types, and individual- and community-level socioeconomic factors. Data were analyzed from January 9 to April 28, 2023.

**Exposures:**

Race and ethnicity of children.

**Main Outcomes and Measures:**

The main outcome was diagnosis of tooth decay in either deciduous or permanent teeth, defined as at least 1 decayed, filled, or missing tooth due to caries. An Anderson-Gill model, a time-to-event model for recurrent tooth decay events with time-varying covariates, stratified by age groups (0-5, 6-10, and 11-18 years) was estimated. A nonlinear multiple additive regression tree–based mediation analysis quantified the relative contributions of factors underlying the observed racial and ethnic disparities.

**Results:**

Among 61 083 children and adolescents aged 0 to 18 years at baseline (mean [SD] age, 9.9 [4.6] years; 30 773 [50.4%] female), 2654 Black individuals (4.3%), 11 213 Hispanic individuals (18.4%), 42 815 White individuals (70.1%), and 4401 individuals who identified as another race (eg, American Indian, Asian, and Hawaiian and Pacific Islander) (7.2%) were identified. Larger racial and ethnic disparities were observed among children aged 0 to 5 years compared with other age groups (Hispanic children: adjusted hazard ratio [aHR], 1.47; 95% CI, 1.40-1.54; Black children: aHR, 1.30; 95% CI, 1.19-1.42; other race children: aHR, 1.39; 95% CI, 1.29-1.49), compared with White children. For children aged 6 to 10 years, higher risk of tooth decay was observed for Black children (aHR, 1.09; 95% CI, 1.01-1.19) and Hispanic children (aHR, 1.12; 95% CI, 1.07-1.18) compared with White children. For adolescents aged 11 to 18 years, a higher risk of tooth decay was observed only in Black adolescents (aHR, 1.17; 95% CI, 1.06-1.30). A mediation analysis revealed that the association of race and ethnicity with time to first tooth decay became negligible, except for Hispanic and children of other race aged 0 to 5 years, suggesting that mediators explained most of the observed disparities. Insurance type explained the largest proportion of the disparity, ranging from 23.4% (95% CI, 19.8%-30.2%) to 78.9% (95% CI, 59.0%-114.1%), followed by dental procedures (receipt of topical fluoride and restorative procedures) and community-level factors (education attainment and Area Deprivation Index).

**Conclusions:**

In this retrospective cohort study, large proportions of disparities in time to first tooth decay associated with race and ethnicity were explained by insurance type and dental procedure types among children and adolescents. These findings can be applied to develop targeted strategies to reduce oral health disparities.

## Key Points

**Question:**

What factors are associated with racial and ethnic differences in the risk of developing tooth decay among US children?

**Findings:**

This cohort study of 61 083 children and adolescents found that compared with White children, all other racial and ethnic groups among those aged 0 to 5 years, Hispanic and Black children among those aged 6 to 10 years, and Black adolescents among those aged 11 to 18 years were at a higher risk of tooth decay. Mediation analysis revealed that individual- and community-level factors, including insurance and dental procedure types, explained most racial and ethnic disparities in the risk of tooth decay.

**Meaning:**

These findings suggest that efforts to reduce racial and ethnic disparities in tooth decay should target different individual- and community-level factors, depending on age and racial and ethnic group.

## Teaser

This cohort study assesses racial and ethnic disparities in the risk of developing tooth decay and evaluates relative contributions of factors mediating the observed disparities among US children.

## Introduction

Tooth decay (caries) is the most common chronic disease among US children yet is often neglected, leading to substantial decreases in children’s quality of life and up to 10 million missed school days.^1,2^ Dental care is one of the greatest unmet children’s health needs, with wide racial and ethnic disparities existing among racially and ethnically minoritized populations, such as American Indian, Asian, Black, Hispanic, Hawaiian and Pacific Islander communities.^3^ Despite efforts to increase dental care utilization among minoritized populations, large oral health disparities remain, with Black and Hispanic children having the poorest oral health of any racial or ethnic groups in the US.^4,5,6,7^ In addition to lack of access to recommended care, individual health behaviors and community-related structural factors also contribute to the high risk of tooth decay in minoritized populations.^5,8^

The influences of racial and ethnic disparities and mediating factors on oral health outcomes are poorly characterized. Previous studies have evaluated the associations of socioeconomic factors with oral health outcomes and disparities.^9,10^ Some studies have reported that racial and ethnic oral health disparities are driven by sociodemographic and demographic factors, although the results have been mixed.^11,12,13,14,15^ While these studies assessed the role of socioeconomic factors on oral health outcomes, none incorporated the associations of race and ethnicity with these socioeconomic factors nor assessed the role of the quality of dental care. Community-level factors, such as rural areas with limited access to care, have been suggested to be associated with oral health disparities.^15^ However, little is known of the relative contribution of individual (socioeconomic and quality of dental care) and community context to the disparities.

Mediation analysis is used to differentiate a third-variable (eg, mediator or confounder) effect that intermediates an observed association between an exposure variable and an outcome variable.^16,17,18^ To determine whether the racial and ethnic disparities in oral health outcomes are associated with differences in socioeconomic factors or quality of care, or from the various living and working environments among different racial and ethnic groups, differentiation and quantification of the mediation of multiple risk factors on racial and ethnic disparities would be warranted. Understanding factors that may mediate the associations of race and ethnicity with oral health outcomes would guide intervention strategies for specific population subgroups. Consequently, we sought to identify racial and ethnic age groups experiencing oral health disparities and determine whether socioeconomic, quality of dental care, and community-level factors mediate the associations of race and ethnicity with disparities in the risk of developing tooth decay among US children.

## Methods

This cohort study was approved by the institutional review board of the Harvard Medical School. Informed consent was waived because the study used only deidentified data. This study followed the Strengthening the Reporting of Observational Studies in Epidemiology (STROBE) and Guideline for Reporting Mediation Analysis–Short Form version (AGReMA-SF) reporting guideline.

### Study Design and Study Population

This retrospective cohort study was conducted using deidentified electronic health record (EHR) data of children and adolescents who enrolled in and received care at a large dental accountable care organization with more than 50 dental offices in Washington, Oregon, and Idaho. The cohort included 61 083 children and adolescents (age 0-18 years) whose baseline (initial assessment) visits were between January 1, 2014, and December 31, 2018, and followed up until December 31, 2020, with at least 1 follow-up visit since the baseline visit. Patients with more than 6-month eligibility gaps during the study period, had self-identified race and ethnicity as multiracial, or noted unspecified sex were excluded from the analysis (eAppendix 1 in Supplement 1). The EHR data contained information on patient demographics, medical history, clinical assessments for oral health conditions (diagnostic, caries risk assessment, and procedure codes), and geographic location.

### Measures

Our primary outcome was diagnosis of tooth decay in either deciduous or permanent teeth, defined as at least 1 decayed, filled, or missing tooth due to caries, assessed by qualified dentists. Recurrences of tooth decay were captured using the longitudinal data for each individual. Individual-level variables included patients’ demographic characteristics (age at visit, sex, race and ethnicity, insurance type), comorbid medical conditions, smoking status, and dental procedures performed within the past 12 months (binary indicators for cleaning, topical fluoride application, sealant, restorative, and extraction) (eTable 1 in Supplement 1). Patient race and ethnicity were self-reported by the patients or guardians and classified as Black, Hispanic, White, and other (including American Indian, Asian, and Hawaiian or Pacific Islander). Insurance status was obtained at each visit and categorized as commercially insured or public (Medicaid or Children’s Health Insurance Program [CHIP]). For dental procedures, preventive (cleaning, sealant, topical fluoride application) and treatment following tooth decay (restorative and extraction) procedures were included. Among preventive procedures, topical fluoride was recommended for those with moderate or high caries risk assessment records at each visit according to the guideline.^19^ Because access to dental care and oral health outcomes vary by socioeconomic factors that can be captured at the community level, Zip Code Tabulation Area (ZCTA)–level factors were considered in the analysis.^20^ ZCTAs are generalized area representations of United States Postal Service zip code service areas.^20^ Community dwelling was determined by the patient zip code reported in the system. ZCTA-level variables were constructed using the American Community Survey data from US Census Bureau and Area Deprivation Index (ADI) database: proportions of all ages that are White, children in each household, unemployed adults, population in poverty, population with less than a high school diploma, not speaking English well, and ADI ranking (0 to 100 percentiles, with 100 indicating the most disadvantaged group).^21,22^ Associations of variables with race and ethnicity were tested with χ^2^ test for categorical variables and Kruskal-Wallis test for continuous variables.

### Statistical Analysis

#### Time-to-Event Analysis

We first estimated Kaplan-Meier curves for the cumulative incidence of first tooth decay events and Nelson-Aalen estimates for the cumulative hazard (accumulated tooth decay events) by racial and ethnic groups. Then, examining all tooth decay events for an individual, we measured racial and ethnic disparities in the risk of developing tooth decay using an Anderson-Gill model, which generalizes the Cox model for recurrent time-to-event adjusting for time-varying covariates.^23^ Based on the Institute of Medicine (IOM) definition of a disparity (difference between different racial and ethnic groups that is not justified by the underlying health conditions or preferences),^24^ we included patients’ unmodifiable demographic characteristics (age at visit, sex, and race and ethnicity) and comorbid medical conditions (growth or developmental problem, musculoskeletal or connective tissue disorder, and neurologic or nerve problem) in the model (model A) to measure racial and ethnic disparities in tooth decay.

We also computed the residual direct effect (RDE) of race and ethnicity, interpreted as the unmediated association of race and ethnicity after adjusting for all other measured covariates; the RDE represents a disparity based on the estimated effect of the race and ethnicity only, thus effectively adjusting for all available variables other than race and ethnicity.^25^ Elastic net regularization was used to select variables for the time-to-event models, using 10-fold internal cross-validation to minimize the risk of overfitting (eAppendix 2 in Supplement 1). Elastic net is a machine learning approach designed to select models in the context of collinearity, which often leads to unstable estimates from traditional stepwise selection approaches.^26,27^ From this approach, we included all individual-level variables that may explain racial and ethnic disparities in the risk of tooth decay (model B). Zip code–level variables were then included into the model with individual-level variables (model C). We used the Greenwood-D’Agostino-Nam test to assess calibration.^28^

As dental care needs and effectiveness may vary with the child’s age,^29^ we performed our analyses separately for the following age groups: 0 to 5, 6 to 10, and 11 to 18 years at initial visits. Observations were censored 5 years from the date of the initial assessment visit. All individual variables were allowed to vary over the follow-up period except for sex and race and ethnicity. Information collected for individuals was aggregated to month level. Missing data were imputed with multiple imputation with chained equation (eAppendix 1 in Supplement 1),^30,31^ and model estimates from 5 imputed data sets were pooled using Rubin rule.^32^

#### Mediation Analysis

For racial and ethnic and age groups that experienced disparities based on the IOM definition (model A), we used mediation analysis to distinguish between direct effect (the association of the variable with the outcome absent the mediator) from the indirect effect (the association of the variable with the outcome that works through the mediator) of a variable with outcome.^33^ Variables excluding age, sex, and comorbid medical conditions were considered as potential factors that mediate the associations of race and ethnicity with tooth decay. We declared a variable a mediator if the variable was significantly associated with the race and ethnicity (exposure) and with diagnosis of tooth decay (outcome), given that all other related factors were included in the model (eAppendix 3 in Supplement 1). Formal mediation analysis was implemented using the mma package in R statistical software version 3.6.1 (R Project for Statistical Computing) for first tooth decay events, incorporating time-varying covariates.^34^ Mediations were modeled using multiple additive regression trees (nonparametric approach). Relative effects of mediators were calculated as the percentage of the total effect associated with corresponding indirect or direct effects from mediation analyses. Positive indirect effects would indicate that race and ethnicity is positively associated with tooth decay through its associations with mediators, and vice versa for negative indirect effects.

No adjustments for multiplicity were implemented. All analyses were performed using R software. *P* values were 2-sided, and statistical significance was set at *P* = .05. Data were analyzed from January 9 to April 28, 2023.

## Results

**Figure 1.**
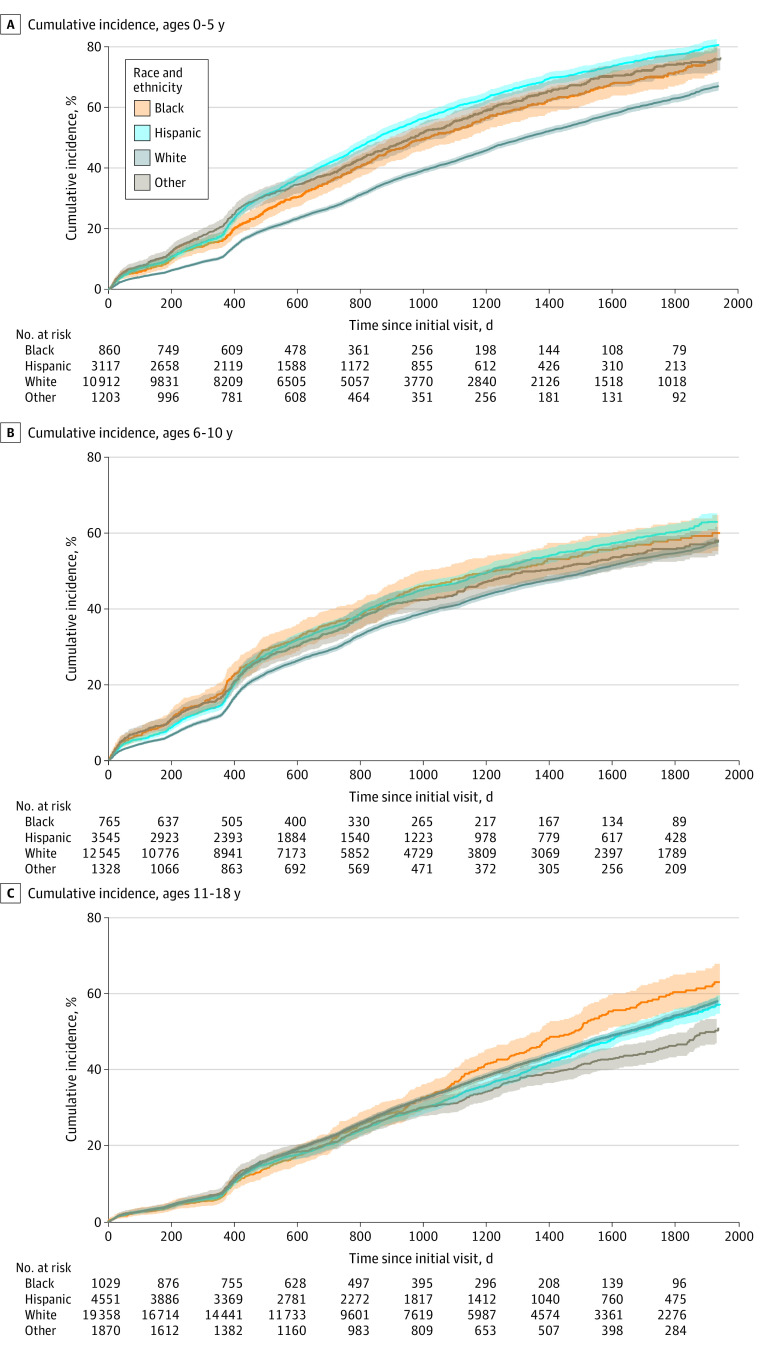
Cumulative Incidence of Tooth Decay by Race and Ethnicity and Age Group The other racial and ethnic group included Asian, American Indian, and Hawaiian or Pacific Islander children and adolescents. Shaded areas represent 95% CIs.

A total of 61 803 children and adolescents (mean [SD] age, 9.9 [4.6] years; 30 773 [50.4%] female) were included in the analysis (Table 1). There were 2654 Black individuals (4.3%), 11 213 Hispanic individuals (18.4%), 42 815 White individuals (70.1%), and 4401 individuals who identified as another race (7.2%). Individual- and community-level variables were significantly different across racial and ethnic groups, except for the sex ratio. The overall 5-year risk of ever experiencing tooth decay was 58.3% (95% CI, 57.7%-58.8%), and the mean number of caries was 1.22 (95% CI, 1.20-1.24) caries per individual (eFigure 1 in Supplement 1). Among children aged 0 to 5 years, 67.5% (95% CI, 66.5%-68.6%) experienced caries, with a mean of 1.69 (95% CI, 1.66-1.72) caries each. Compared with White children, Black children (hazard ratio [HR], 1.38; 95% CI, 1.27-1.50), Hispanic children (HR, 1.49; 95% CI, 1.42-156), and children who identified as another race (HR, 1.41; 95% CI, 1.31-1.52) were at higher risk of tooth decay (Figure 1; eFigure 2 in Supplement 1). Among children aged 6 to 10 years, 56.2% (95% CI, 55.3%-57.2%) of children experienced tooth decay, with a mean of 1.08 (95% CI, 1.06-1.10) caries each. Among these children and compared with White children, Black children (HR, 1.13; 95% CI, 1.02-1.25) and Hispanic children (HR, 1.10; 95% CI, 1.14-1.16) were at higher risk of tooth decay. For individuals aged 11 to 18 years, 54.3% (95% CI, 55.2%-53.4%) experienced tooth decay, with a mean of 1.06 (95% CI, 1.05-1.08) caries each, and only Black adolescents were at a higher risk (HR, 1.14; 95% CI, 1.03-1.26).

**Table 1.** Baseline Characteristics of the Study Population

Characteristic	Individuals, No. (%)					*P* value
	Overall (N = 61 083)	Black (n = 2654)	Hispanic (n = 11 213)	White (n = 42 815)	Other (n = 4401)^a^	
Age, y						
Mean (SD)	9.48 (5.01)	8.85 (5.21)	9.16 (4.95)	9.62 (5.01)	9.35 (5.03)	<.001
<6	16 092 (26.3)	860 (32.4)	3117 (27.8)	10 912 (25.5)	1203 (27.3)	<.001
6-10	18 183 (29.8)	765 (28.8)	3545 (31.6)	12 545 (29.3)	1328 (30.2)	
11-18	26 808 (43.9)	1029 (38.8)	4551 (40.6)	19 358 (45.2)	1870 (42.5)	
Sex						
Male	30 310 (49.6)	1316 (49.6)	5546 (49.5)	21 318 (49.8)	2130 (48.4)	.36
Female	30 773 (50.4)	1338 (50.4)	5667 (50.5)	21 497 (50.2)	2271 (51.6)	
Public insurance	26 560 (43.5)	1887 (71.1)	7472 (66.6)	14 993 (35.0)	2208 (50.2)	<.001
Smoking status	1111 (1.8)	31 (1.2)	110 (1.0)	916 (2.1)	54 (1.2)	<.001
Medical condition						
Growth or development	2959 (4.8)	138 (5.2)	392 (3.5)	2265 (5.3)	164 (3.7)	<.001
Neurologic or nerve	3439 (5.6)	129 (4.9)	397 (3.5)	2743 (6.4)	170 (3.9)	<.001
Musculoskeletal or connective tissue disorder	378 (0.6)	8 (0.3)	40 (0.4)	310 (0.7)	20 (0.5)	<.001
Dental procedures^b^						
Cleaning	50 787 (83.1)	2153 (81.1)	9411 (83.9)	35 492 (82.9)	2087 (84.8)	<.001
Fluoride	43 839 (71.8)	1881 (70.9)	8569 (76.4)	30 104 (70.3)	3285 (74.6)	<.001
Sealant	21 975 (36.0)	900 (33.9)	4313 (38.5)	15 145 (35.4)	1617 (36.7)	<.001
Restorative	18 345 (30.0)	802 (30.2)	3817 (34.0)	12 338 (28.8)	1388 (31.5)	<.001
Extraction	6816 (11.2)	302 (11.4)	1466 (13.1)	4493 (10.5)	555 (12.6)	<.001
Community-level variables, mean (SD)						
Population with <high school diploma, %	10.01 (5.98)	10.81 (6.32)	12.36 (6.90)	9.35 (5.48)	9.98 (6.28)	<.001
Area Deprivation Index	34.92 (16.21)	31.83 (13.92)	37.83 (15.82)	34.77 (16.43)	30.79 (14.91)	<.001

^a^ Includes children and adolescents who identified as Asian, American Indian, and Hawaiian or Pacific Islander.

^b^ Procedures performed between January 1, 2014, and December 31, 2018 (baseline enrollment period).

### Oral Health Disparities

**Figure 2.**
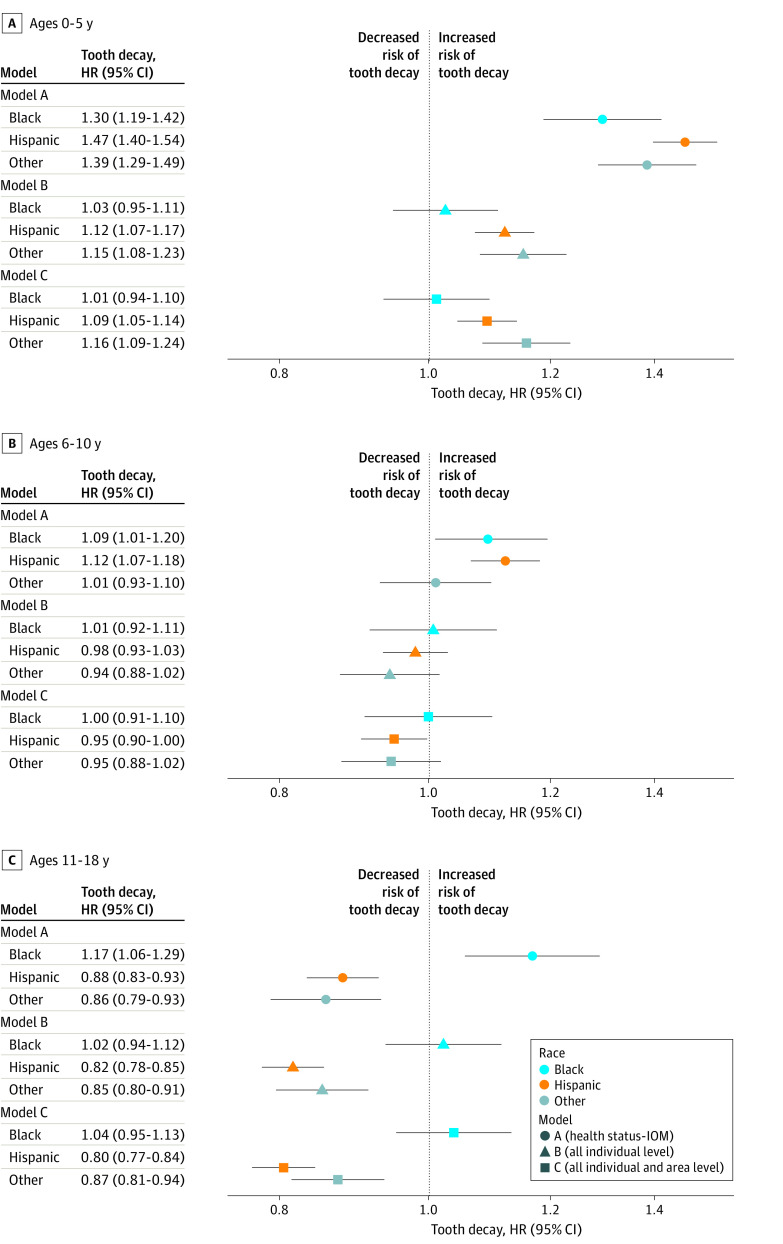
Results of Time to Tooth Decay Regression Models The reference group for the hazard ratio (HR) estimates was White. The other racial and ethnic group included Asian, American Indian, and Hawaiian or Pacific Islander children and adolescents. Error bars indicate 95% CIs; IOM, Institute of Medicine.

Following the IOM framework of measuring disparity, when we adjusted for health status (age, sex, and comorbid medical conditions), all racially and ethnically minoritized groups among children aged 0 to 5 years experienced significantly higher risk of tooth decay compared with White children (Black children: adjusted HR [aHR], 1.30; 95% CI, 1.19-1.42; Hispanic children: aHR, 1.47; 95% CI, 1.40-1.54; children with other race: aHR, 1.39; 95% CI, 1.29-1.49) (Figure 2; eTable 2 in the Supplement). Among children aged 6 to 10 years and compared with White children, Black children (aHR, 1.09; 95% CI, 1.01-1.19) and Hispanic children (aHR, 1.12; 95% CI, 1.07-1.18) were at higher risk of tooth decay. Among adolescents aged 11 to 18 years, only Black adolescents experienced a higher risk of tooth decay (aHR, 1.17; 95% CI, 1.06-1.30).

Using elastic net regularization, insurance type (commercially insured vs Medicaid or CHIP), smoking status, dental procedures (cleaning, topical fluoride application, sealant, restorative, and extraction) were selected among individual-level variables (eAppendix 2 in Supplement 1). For community-level variables, education attainment (proportion of population with less than a high school diploma) and ADI were selected. When all selected individual-level factors were adjusted (model B), Black children aged 0 to 5 years no longer experienced a higher risk of tooth decay compared with White children (Figure 2). For children aged 6 to 10 years and adolescents aged 11 to 18 years, none of the racially or ethnically minoritized groups experienced higher risk of tooth decay than White children and adolescents. Insurance type (children aged 0-5 years: aHR, 1.38; 95% CI, 1.33-1.44; children aged 6-10 years: aHR, 1.24; 95% CI, 1.19-1.29; adolescents aged 11-18 years: aHR, 1.35; 95% CI, 1.30-1.40) and dental procedures were associated with risk of tooth decay (eTable 2 in Supplement 1).

With the inclusion of community-level factors (model C), zip code–level education attainment and ADI were associated with risk of tooth decay for children aged between 0 and 10 years (eTable 2 in Supplement 1). For adolescents, ADI was the only community-level variable associated with the risk of tooth decay. When all potential mediators (insurance type, smoking status, types of dental procedure received, and community-level factors) were included in the model, the model had a concordance statistic of 0.71 and passed Greenwood-D’Agostino-Nam test (eFigure 3 in the Supplement).^28^

### Mediators of Time to First Tooth Decay

**Figure 3.**
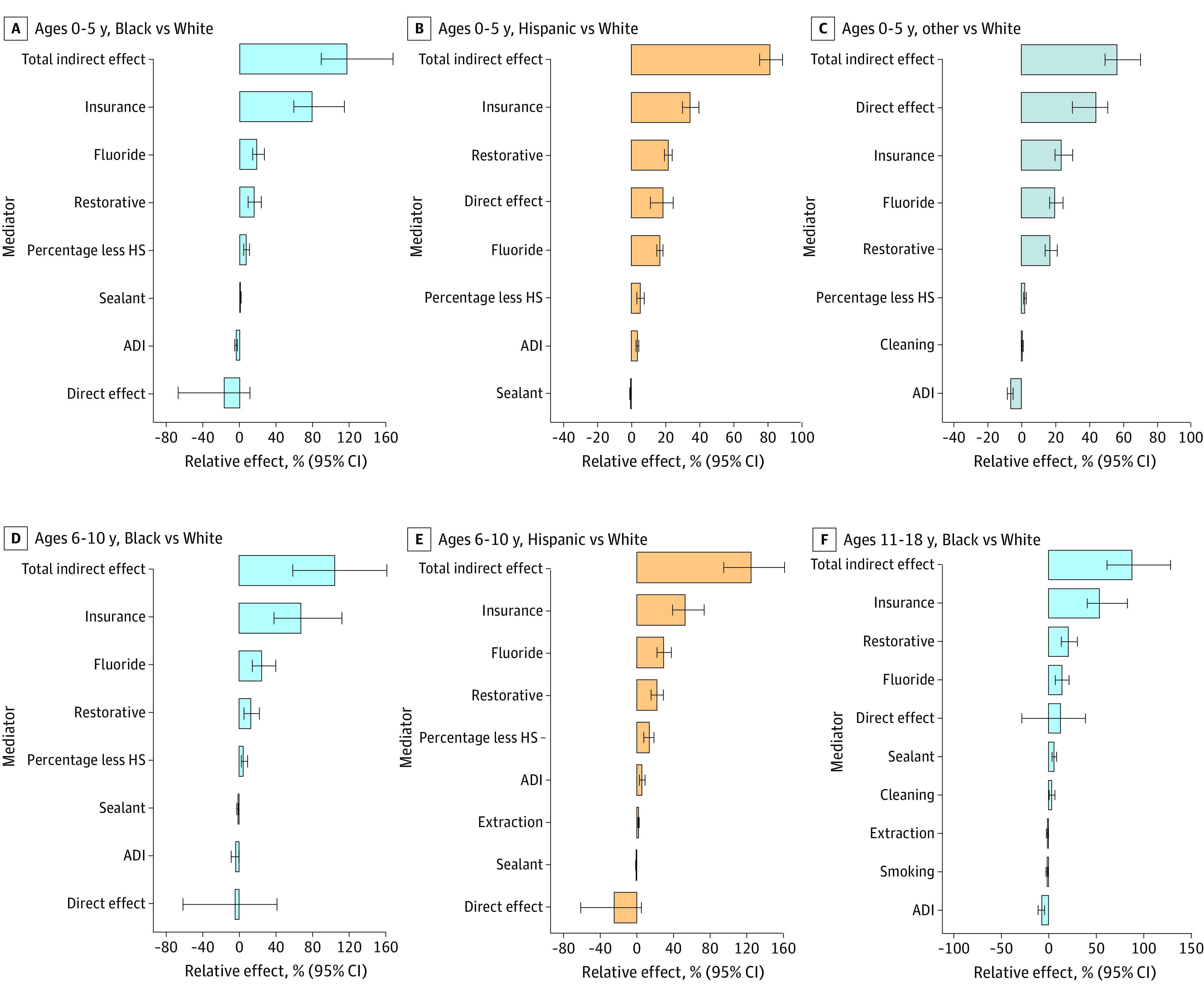
Relative Effects From Mediation Analysis for Time to First Tooth Decay The other racial and ethnic group included Asian, American Indian, and Hawaiian or Pacific Islander children and adolescents. Error bars indicate 95% CIs. ADI indicates Area Deprivation Index; HS, high school.

Mediation analysis was performed among groups that experienced disparities in model A (all minoritized racial and ethnic groups among children aged 0-5 years, Black and Hispanic children aged 6-10 years, and Black adolescents aged 11-18 years) and found that individual- and community-level factors accounted for substantial proportions of observed racial and ethnic disparities in the risk of tooth decay; the direct associations of race and ethnicity with time to first tooth decay became negligible, suggesting that mediators explained most of the observed disparities except for those observed for Hispanic individuals (unexplained: 18.6%; 95% CI, 11.2%-24.6%) and those who identified as another race (unexplained: 43.8%; 95% CI, 30.0%-50.8%) compared with White children aged 0 to 5 years (Figure 3 and Table 2; eTable 3 in Supplement 1). Variation in insurance type explained the largest proportions of the associations of race and ethnicity with tooth decay risk, ranging from 23.4% (95% CI, 19.8%-30.2%) to 78.9% (95% CI, 59.0%-114.1%) of the disparities, followed by receipt of topical fluoride and restorative procedures prior to the diagnosis of tooth decay (Figure 3 and Table 2). Community-level ADI also explained shares of racial and ethnic disparities for all age groups, and educational attainment explained disparities among those aged 0 to 5 years and 6 to 10 years.

**Table 2.** Mediation Analysis

Variable	RE, % (95% CI)^a^					
	Age 0-5 y			Age 6-10 y		Age 11-18 y
	Black vs White	Hispanic vs White	Other vs White^b^	Black vs White	Hispanic vs White	Black vs White
Total direct effect	−16.9 (−67.2 to 11.1)	18.6 (11.2 to 24.6)	43.8 (30.0 to 50.8)	−4.3 (−61.2 to 41.4)	−24.7 (−61.2 to 5.1)	12.5 (−28.0 to 38.7)
Total indirect effect	116.9 (88.9 to 167.2)	81.4 (75.4 to 88.8)	56.2 (49.2 to 70.0)	104.3 (58.6 to 161.2)	124.7 (94.9 to 161.2)	87.5 (61.3 to 128.0)
Insurance	78.9 (59.0 to 114.1)	34.5 (30.1 to 39.7)	23.4 (19.8 to 30.2)	67.4 (38.1 to 112.2)	52.7 (39.0 to 73.5)	53.4 (40.6 to 82.7)
Fluoride application	18.5 (14.1 to 26.9)	16.8 (15.0 to 18.6)	19.6 (16.6 to 24.5)	24.6 (14.5 to 40.0)	29.3 (22.1 to 37.7)	14.1 (7.1 to 21.5)
Restorative	15.7 (9.1 to 23.5)	21.7 (19.5 to 24.0)	16.8 (14.0 to 21.1)	12.8 (5.4 to 22.2)	22.0 (15.6 to 29.0)	20.7 (13.4 to 30.3)
Cleaning	NA^c^	NA^c^	0.6 (0.2 to 1.2)	0.0 (0.0 to 0.0)	0.0 (0.0 to 0.0)	3.3 (0.7 to 6.7)
Sealant	0.7 (0.2 to 1.5)	−0.5 (−0.9 to −0.2)	NA^c^	−1.2 (−2.5 to −0.5)	−0.7 (−1.2 to −0.3)	5.7 (3.7 to 8.5)
Extraction	NA^c^	NA^c^	3.3 (2.1 to 4.7)	0.0 (0.0 to 0.0)	1.9 (1.2 to 2.7)	−1.2 (−2.3 to −0.5)
Smoking status	NA^c^	NA^c^	NA	0.0 (0.0 to 0.0)	0.0 (0.0 to 0.0)	−1.5 (−2.7 to −0.7)
Proportion of population with <high school	7.0 (4.3 to 10.6)	5.3 (3.3 to 7.6)	2.0 (1.3 to 2.9)	4.5 (2.4 to 9.3)	13.7 (7.6 to 18.7)	0.0 (0.0 to 0.0)
Area Deprivation Index	−3.8 (−5.6 to −2.6)	3.6 (2.8 to 4.4)	−6.2 (−8.2 to −4.8)	−3.7 (−8.6 to −0.1)	5.6 (2.9 to 9.0)	−7.1 (−11.0 to −4.2)

Abbreviations: NA, not applicable; RE, relative effect.

^a^ Mediation analyses were performed among groups that experienced disparities in tooth decay following the Institute of Medicine framework of measuring disparity (with adjustment for unmodifiable demographics and health status; age, sex, and comorbid medical conditions). The REs were calculated as the percentage of the total effect associated with corresponding indirect or direct effects from mediation analyses. Positive indirect effects would indicate that race or ethnicity was positively associated with tooth decay through its association with mediators, and vice versa for negative indirect effects.

^b^ Includes Asian, American Indian, and Hawaiian or Pacific Islander.

^c^ Variable not selected as mediators (eAppendix 3 in the Supplement) but included as covariates. Relative effects were calculated among mediators.

## Discussion

In this retrospective cohort study, we observed that all racially and ethnically minoritized groups of children aged 0 to 5 years were at a higher risk of tooth decay compared with White children. For school-aged children (age 6-10 years), disparities were observed among Black and Hispanic children, and for adolescents aged 11 to 18 years, only Black adolescents experienced a higher risk of tooth decay compared with White adolescents. A mediation analysis further revealed that insurance type, receipt of certain dental procedures, and community-level socioeconomic factors, including education attainment and ADI, explained most of the observed racial and ethnic disparities in tooth decay risk.

While Medicaid and the CHIP meets its equal access requirement for dental care for children, our study found that insurance type mediated the largest percentage of racial and ethnic disparities in tooth decay after accounting for the receipt of dental procedures (access to care for both preventive and problem-oriented visits) and community-level socioeconomic factors (education attainment and ADI). This finding is consistent with a 2016 study by Shariff and Edelstein^5^ that found no differences between use of dental care between publicly and privately insured children but poorer oral health status among publicly insured children. Due to lack of information on other socioeconomic status indicators in our EHR data, insurance type served as a proxy for individual socioeconomic status, as it has in previous studies.^35,36,37^ Thus, our findings may suggest that differences in individual socioeconomic status between publicly and privately insured children, such as family income and food insecurity, could contribute to poor oral health outcomes. It could also suggest that benefits covered by Medicaid and the CHIP may not provide sufficient care to meet publicly insured children’s needs or to reduce the progression of tooth decay.^5^ Therefore, the association of insurance status with oral health disparities is multifaceted; equity in the use of dental care between Medicaid or the CHIP and privately insured children may not result in equity in children’s oral health.

The evidence-based guideline recommends professional topical fluoride treatments for individuals at elevated risk (ie, moderate or high) of tooth decay based on caries-risk assessment.^38^ While fluoride has been proven to prevent tooth decay by making the enamel more resistant to the action of acids,^39^ topical fluoride application was positively associated with the risk of developing tooth decay in our analysis because it was only applied to children at elevated risk of caries according to the guideline.^38^ Caries risk assessment is part of a comprehensive treatment plan approach and involves estimating children at low, moderate, and high risk of caries based on a child’s age; social, behavioral, and medical risk factors (eg, low health literacy, high sugar consumption, special health care needs); protective factors (ie, tooth brushing frequency, exposure to fluoride); and clinical findings (ie, visible plaque on teeth, presence of enamel defects).^19^ Thus, receipt of topical fluoride, in part, indicates social and behavioral risk factors of children and how these factors may mediate the racial and ethnic disparities.

Our findings can help to inform targeted efforts to reduce racial and ethnic disparities in oral health outcomes among children. Through a mediation analysis, we demonstrated that race and ethnicity could be viewed as a social construct in which health disparities were mediated through socioeconomic and geographic differences rather than through biological differences between racial and ethnic groups.^40,41,42^ Initiatives to emphasize the clinical significance of socioeconomic and behavior risk factors could help to reduce oral health disparities. Refinements in Medicaid and the CHIP policy or oral health promotion interventions targeted for Medicaid-eligible families may have potential to reduce oral health disparities by race and ethnicity.

### Limitations

Our study has limitations. First, the mediation analysis we performed is constrained by the candidate mediators that were selected for this retrospective analysis. We could not account for potential unmeasured factors, such as individual-level socioeconomic, behavioral, and structural risk factors, which may introduce a possibility of missed mediation. Hence, our study may not imply causal mediation effect of race and ethnicity or provide a comprehensive assessment of all the possible mechanisms associated with underlying racial and ethnic disparities in oral health. Along with some sociodemographic factors, patient perception (eg, dental anxiety) also influences the receipt of specific dental procedures, which could not be incorporated in our analysis. Future studies are warranted to directly address other potential mediators. Additionally, because our study sample was a convenience sample of children who were enrolled and received care at large dental accountable care organizations across 3 states, the findings may not be generalizable to beyond these populations. Furthermore, while we incorporated community-level factors in the analysis, we did not account for the intracluster correlations, and we may have overstated the precision of some estimates. Because these community-level variables were obtained from 2014 to 2018 American Community Survey 5-year data to represent community-level characteristics of individuals at their baseline visits (2014-2018), there may be temporal misalignment between the outcome and the community-level characteristics during the follow-up period of these individuals. Moreover, these variables do not fully capture the information from individual-level socioeconomic status (eg, household income level).

## Conclusion

This cohort study found that individual- and community-level socioeconomic factors (insurance type, community-level educational attainment, and ADI) and receipt of dental procedures explained a large share of the observed racial and ethnic disparity in the risk of tooth decay among racially and ethnically minoritized children and adolescents (Black individuals, Hispanic individuals, and individuals who identified as another race) compared with White children and adolescents. These findings provide insights in identifying specific factors within racial and ethnic groups that could be targeted for intervention strategies. Providing additional resources to these population subgroups may have the potential to improve oral health outcomes and reduce oral health disparities.

## Notes

Supplement 1. **eAppendix 1.** Participant Recruitment Flowchart and Missing Data **eAppendix 2.** Time-to-Event Model Selection **eAppendix 3.** Mediation Analysis **eTable 1.** Dental Procedure Codes Used for Procedure Grouping and Baseline Caries Risk **eTable 2.** Results of Time-to-Event Models: Time to Tooth Decay Events (First and Recurrent) **eTable 3.** Mediation Analysis Results: Time to First Tooth Decay Event **eFigure 1.** Cumulative Incidence and Cumulative Hazard of Tooth Decay by Race and Ethnicity Among Overall Population **eFigure 2.** Cumulative Hazard of Tooth Decay by Race and Ethnicity and Age Groups **eFigure 3.** Validity of the Time-to-Event Model
eReferences

Supplement 2.
Data Sharing Statement
