# Supplementary material for: Analysis of Race and Ethnicity, Socioeconomic Factors, and Tooth Decay Among US Children
Source: JAMA Netw Open. 2023 Jun 15;6(6):e2318425. doi: 10.1001/jamanetworkopen.2023.18425 (PMC10273024; doi:10.1001/jamanetworkopen.2023.18425)
Supplement: Supplement 1. — eAppendix 1. Participant Recruitment Flowchart and Missing Data eAppendix 2. Time-to-Event Model Selection eAppendix 3. Mediation Analysis eTable 1. Dental Procedure Codes Used for Procedure Grouping and Baseline Caries Risk eTable 2. Results of Time-to-Event Models: Time to Tooth Decay Events (First and Recurrent) eTable 3. Mediation Analysis Results: Time to First Tooth Decay Event eFigure 1. Cumulative Incidence and Cumulative Hazard of Tooth Decay by Race and Ethnicity Among Overall Population eFigure 2. Cumulative Hazard of Tooth Decay by Race and Ethnicity and Age Groups eFigure 3. Validity of the Time-to-Event Model eReferences [file jamanetwopen-e2318425-s001.pdf]

Supplemental Online Content

Choi SE, White J, Mertz E, Normand SL. Analysis of race and ethnicity, socioeconomic factors, and tooth decay among US children. *JAMA Netw Open*. 2023;6(6):e2318425. doi:10.1001/jamanetworkopen.2023.18425

eAppendix 1. Participant Recruitment Flowchart and Missing Data
eAppendix 2. Time-to-Event Model Selection
eAppendix 3. Mediation Analysis
eTable 1. Dental Procedure Codes Used for Procedure Grouping and Baseline Caries Risk
eTable 2. Results of Time-to-Event Models: Time to Tooth Decay Events (First and Recurrent)
eTable 3. Mediation Analysis Results: Time to First Tooth Decay Event
eFigure 1. Cumulative Incidence and Cumulative Hazard of Tooth Decay by Race and Ethnicity Among Overall Population
eFigure 2. Cumulative Hazard of Tooth Decay by Race and Ethnicity and Age Groups
eFigure 3. Validity of the Time-to-Event Model
eReferences

This supplemental material has been provided by the authors to give readers additional information about their work.

© 2023 Choi SE et al. *JAMA Network Open.*

eAppendix 1. Participant Recruitment Flowchart and Missing Data

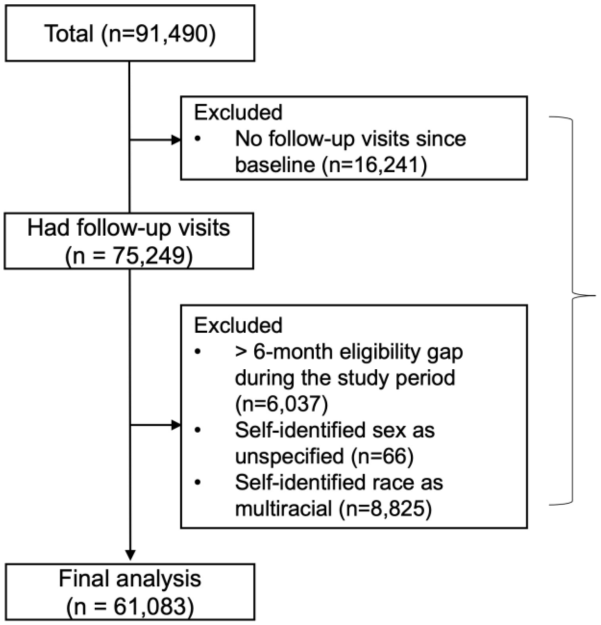

Racial distribution of excluded patients

	White	Hispanic	Black	Asian	Other	Multiracial
Total (n=94,190)	56643 (61.9%)	14714 (16.1%)	3513 (3.8%)	3089 (3.4%)	2534 (2.8%)	10997 (12.0%)
No follow-up since baseline (n=16,241)	10166 (62.6%)	2266 (13.9%)	610 (3.8%)	426 (2.6%)	412 (2.5%)	2361 (14.5%)
> 6-month eligibility gap (n=6,037)	3519 (58.2%)	1153 (19.1%)	278 (4.6%)	153 (2.5%)	187 (3.1%)	747 (12.4%)
Unspecified sex (n=66)	51 (77.3%)	4 (6.1%)	0 (0.0%)	2 (3.0%)	3 (4.5%)	6 (9.1%)

Variables in the below table had missing data, which were imputed with multiple imputation with chained equation.[1,2] Result estimates from five imputed datasets were pooled using Rubin's rule.[3]

Variable	% Missing
Race	14.59
Neurologic/Nerve problem	0.03
Musculoskeletal/Connective tissue disorder	0.03
Growth/Development delay	0.03
Smoke	0.08
Dental procedure - Cleaning	0.10
Dental procedure - Fluoride application	0.10
Dental procedure - Sealant	0.10
Dental procedure - Restorative	0.10
Dental procedure - Extraction	0.10
Percentage of less than high school education	0.01

© 2023 Choi SE et al. *JAMA Network Open.*

eAppendix 2. Time-to-Event Model Selection

Elastic net regularization seeks to select predictors from a set of candidate variables with the goal of generating parsimonious models by minimizing overfitting, while preserving a high degree of predictive power, assessed through repeated internal cross-validation.[4,5] It utilizes a combination of ridge regression and lasso regression. Ridge regression shrinks coefficients of correlated predictors towards each other, while lasso regression penalizes nonzero regression coefficients, choosing one correlated predictor and discarding the others. Elastic net mixes ridge and lasso regression by adjusting the penalty parameter to balance the two methods.

In our study, this method was implemented using the glmnet package in the statistical program R.[4,6] The glmnet algorithm uses cyclical coordinate descent, which successively optimizes the objective function over each parameter with others fixed, and cycles repeatedly until convergence over the course of 10-fold repeated internal cross-validation.

Each observation has the following tuple, $(t_i^l, t_i^u, \delta_i, x_i)$: t_i^l and t_i^u are the time periods in which the patient i had this covariate information, δ_i (an indicator of whether the event happened to the patient at t_i^u and x_i (patient's vector of covariate measurements)

$$\text{Partial Likelihood}$$

$$L(\boldsymbol{\beta}) = \prod_{i=1}^{N} \left(\frac{e^{\boldsymbol{x}_i^T \boldsymbol{\beta}}}{\sum_{j \in R(t_i)}^{N} e^{\boldsymbol{x}_j^T \boldsymbol{\beta}}} \right)^{\delta_i}$$

$$\text{Partial Log-Likelihood}$$

$$\ell(\boldsymbol{\beta}) = \sum_{i=1}^{N} \delta_i \left\{ \boldsymbol{x}_i^T \boldsymbol{\beta} - \log \left[\sum_{j \in R(t_i)}^{N} \exp(\boldsymbol{x}_j^T \boldsymbol{\beta}) \right] \right\} \quad (1)$$

$R(t_i)$ is the risk-set or index of patients who were alive/non-censored at the event time t_i . The elastic-net model combines a weighted L1 and L2 penalty term of the coefficient vector, the former which can lead to sparsity (i.e. coefficients which are strictly zero) and the latter which ensures smooth coefficient shrinkage. The objective function is as follows,

$$\hat{\boldsymbol{\beta}} = \arg\min_{\boldsymbol{\beta}} \sum_{i=1}^{N} \delta_i \left\{ \boldsymbol{x}_i^T \boldsymbol{\beta} - \log \left[\sum_{j \in R(t_i)}^{N} \exp(\boldsymbol{x}_j^T \boldsymbol{\beta}) \right] \right\} + \lambda \big(\alpha \|\boldsymbol{\beta}\|_1 + 0.5(1-\alpha) \|\boldsymbol{\beta}\|_2 \big) \quad (2)$$

The parameter λ determines the overall level of regularization. In our study, the parameter λ value with the error (between the model and observed outcomes) is within 1 standard error of the cross-validated errors over the course of 10-fold repeated internal cross-validation was chosen as covariates as suggested in Friedman et al.[4]

© 2023 Choi SE et al. *JAMA Network Open.*

Cross-validation plot

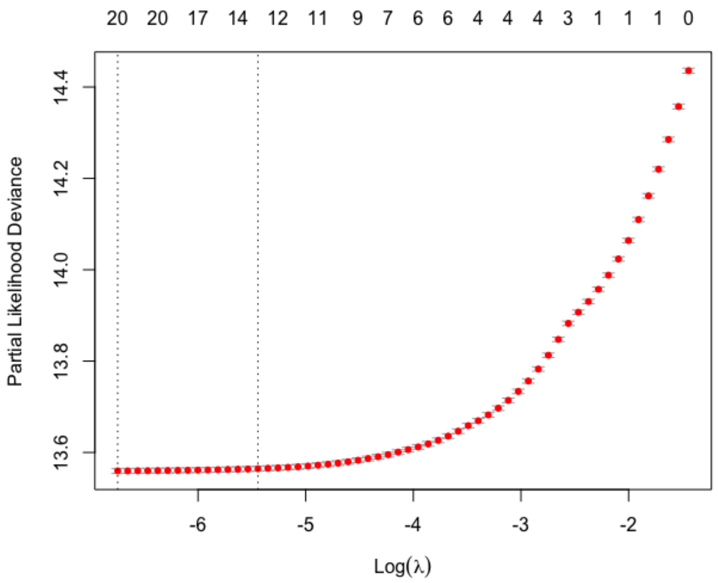

The left vertical line in our plot shows us where the CV-error curve hits its minimum. The right vertical line shows us the most regularized model with CV-error within 1 standard deviation of the minimum. The numbers across the top are the number of nonzero coefficient estimates.

Selected covariates

Variable selection was performed on five imputed datasets; Results were the same except for one dataset in which Neurologic/Nerve problem was not selected. Our final analysis included all selected variables during this process – age at visit, sex, insurance status at visit, race/ethnicity, medical conditions (growth/developmental problem, musculoskeletal/connective tissue disorder, and neurologic/nerve problem), smoking status, dental procedures (cleaning, topical fluoride, sealant, restorative procedures, and extraction), and community-level variables (percentage of individuals with less than high school education and ADI).

```
> coef(cv.fit, s = 'lambda.1se')
27 x 1 sparse Matrix of class "dgCMatrix"
                           1
age_visit        0.703926827
female          -0.008154342
insure_visit    -0.24975819
race_blk         0.188361221
race_hisp        0.118394042
race_other_all   0.175182717
m_cvd            .
m_resp           .
m_diab           .
m_growth         0.074396692
m_eat            .
m_musc           0.034268712
m_neuro          0.021262589
smoke            0.177262980
```

© 2023 Choi SE et al. *JAMA Network Open.*

```
proc_clean_yr   -0.560221472
proc_fluor_yr    0.680005258
proc_seal_yr    -0.112726235
proc_restor_yr   0.783159250
proc_extract_yr  0.162415484
pct_employed     .
pct_child        .
pct_english      .
pct_white        .
pct_lessHS       0.025394460
pct_poverty      .
pct_uninsured    .
ADI_nat         -0.02977071
```

© 2023 Choi SE et al. *JAMA Network Open.*

eAppendix 3. Mediation Analysis

Mediation analysis seeks to identify and explain the mechanism or process that underlies an observed relationship between an independent variable and a dependent variable via the inclusion of a third variable, known as a mediator variable.[7] In this study, we aim to explain the effects of race/ethnicity on the risk of developing dental caries.

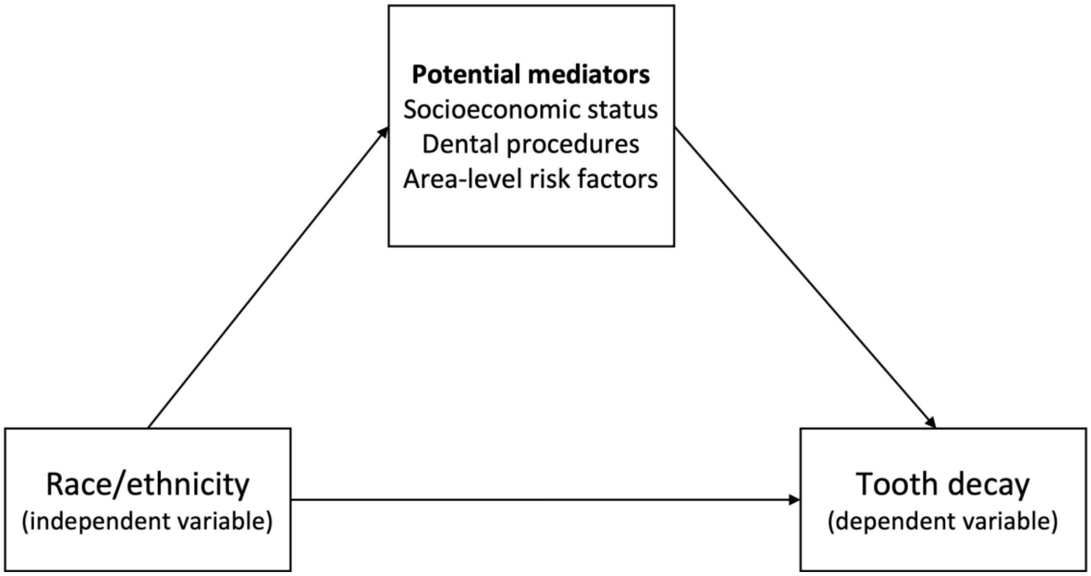

Mediation analysis aims to estimate the average effect of the exposure (race) on the outcome (first tooth decay) while other conditions being held constant.[8] This approach allows us to decompose to the total effect of exposure on the outcome into those contributed by the exposure and those contributed indirectly by the mediators.

First, we identified factors may mediate the relationship between race/ethnicity and outcomes, were identified by testing whether 1) the variable is significantly associated with the outcome and 2 the variable is significantly associated with the predictor, given that all other related factors are included in the model. These conditions were tested using chi-square statistics or analysis of variance for categorical and continuous variables, respectively, with a significance level 0.1 (see below). Next, mediation effects (direct and indirect effects of race/ethnicity on dental caries) were estimated using a nonlinear multiple additive regression tree (MART)-based mediation analysis and 100 bootstrap iterations to account uncertainty of the estimates. MART is used to fit the relationships among variables, based on which inferences on mediation effects can be made taking into consideration more complicated variable transformations and interactions without the necessity of pre-specifying them. Also, when MART is used, all missing data can be handled without losing information.[9] The relative effects were calculated as the proportions of indirect or direct effects out of the total effect. The *mma* software package was used to identify potential mediators and estimate mediation effects.[10]

Selecting mediator variables
Predictors that were not selected as mediators are highlighted in grey below.

Total population

	Black vs White		Hispanic vs White		Other vs White	
	P-value outcome	P-value exposure	P-value outcome	P-value exposure	P-value outcome	P-value exposure
Insurance	0.000	0.000	0.000	0.000	0	0
Smoking	0.000	0.000	0.000	0.000	0	0.049
Cleaning	0.000	0.000	0.000	0.000	0	0.021
Fluoride application	0.000	0.000	0.000	0.000	0	0
Sealant	0.000	0.000	0.000	0.728	0	0.096
Restorative	0.000	0.000	0.000	0.000	0	0
Extraction	0.000	0.857	0.000	0.000	0	0
ADI Index	0.000	0.000	0.000	0.000	0	0.013
% Less than HS	0.000	0.000	0.000	0.000	0	0

Age 0-5

	Black vs White		Hispanic vs White		Other vs White	
	P-value outcome	P-value exposure	P-value outcome	P-value exposure	P-value outcome	P-value exposure
Insurance	0.000	0.000	0.000	0.000	0.000	0.000
Smoking	0.479	NA	0.776	NA	0.775	NA
Cleaning	0.012	0.692	0.000	0.604	0.000	0.002
Fluoride application	0.000	0.000	0.000	0.000	0.000	0.000
Sealant	0.001	0.016	0.000	0.000	0.000	0.371
Restorative	0.000	0.000	0.000	0.000	0.000	0.000
Extraction	0.203	NA	0.180	NA	0.137	NA
ADI Index	0.000	0.000	0.000	0.000	0.000	0.000
% Less than HS	0.000	0.000	0.000	0.000	0.000	0.000

Age 6-10

	Black vs White		Hispanic vs White		Other vs White	
	P-value outcome	P-value exposure	P-value outcome	P-value exposure	P-value outcome	P-value exposure
Insurance	0.000	0.000	0.000	0.000	0.000	0.000
Smoking	0.995	NA	0.957	NA	0.955	NA
Cleaning	0.000	0.128	0.000	0.154	0.000	0.001
Fluoride application	0.000	0.000	0.000	0.000	0.000	0.000
Sealant	0.002	0.005	0.000	0.000	0.002	0.004
Restorative	0.000	0.000	0.010	0.000	0.000	0.458
Extraction	0.000	0.451	0.000	0.000	0.000	0.000
ADI Index	0.013	0.000	0.003	0.000	0.007	0.000
% Less than HS	0.000	0.000	0.000	0.000	0.000	0.000

Age 11-18

	Black vs White		Hispanic vs White		Other vs White	
	P-value outcome	P-value exposure	P-value outcome	P-value exposure	P-value outcome	P-value exposure
Insurance	0.000	0.000	0.000	0.000	0.000	0.000

© 2023 Choi SE et al. *JAMA Network Open.*

Smoking	0.000	0.000	0.000	NA	0.000	0.000
Cleaning	0.000	0.006	0.000	0.170	0.001	0.000
Fluoride application	0.000	0.000	0.000	0.000	0.000	0.491
Sealant	0.000	0.000	0.000	0.000	0.000	0.147
Restorative	0.000	0.000	0.000	0.001	0.000	0.000
Extraction	0.000	0.000	0.022	0.035	0.000	0.110
ADI Index	0.000	0.000	0.448	0.000	0.694	0.000
% Less than HS	0.926	NA	0.000	NA	0.000	NA

© 2023 Choi SE et al. *JAMA Network Open.*

eTable 1. Dental Procedure Codes Used for Procedure Grouping and Baseline Caries Risk

**Grouping**		**CDT procedure codes**
Cleaning		D1110, D1120
Fluoride application		D1206, D1208
Sealant		D1351, D1353
Restorative		D2***
Extraction		D7111, D7140, D7210, D7220, D7230, D7240, D7241, D7250, D7251
Caries risk	Low	D0601
	Moderate	D0602
	High	D0603

© 2023 Choi SE et al. *JAMA Network Open.*

eTable 2. Results of Time-to-Event Models: Time to Tooth Decay Events (First and Recurrent)

Aged 0-5 years

	Model A	Model B	Model C
	HR (95% CI)	HR (95% CI)	HR (95% CI)
Race/ethnicity			
Latino	1.47 (1.40, 1.54)	1.12 (1.07, 1.17)	1.09 (1.05, 1.14)
Black	1.30 (1.19, 1.42)	1.03 (0.95, 1.11)	1.01 (0.94, 1.10)
Other	1.39 (1.29, 1.49)	1.15 (1.08, 1.22)	1.16 (1.09, 1.24)
Age at visit	1.49 (1.42, 1.57)	1.26 (1.20, 1.33)	1.25 (1.19, 1.32)
Age at visit squared	0.95 (0.95, 0.96)	0.96 (0.96, 0.97)	0.96 (0.96, 0.97)
Sex (vs Male)	1.00 (0.96, 1.04)	0.99 (0.96, 1.03)	0.99 (0.96, 1.03)
Medical conditions			
Growth/Development	1.14 (1.01, 1.28)	1.10 (0.99, 1.23)	0.85 (0.61, 1.18)
Musculoskeletal/Connective tissue disorder	0.82 (0.55, 1.22)	0.85 (0.60, 1.20)	1.00 (0.88, 1.14)
Neurologic/Nerve problem	1.03 (0.89, 1.18)	1.01 (0.89, 1.15)	1.10 (0.99, 1.22)
Insurance type (vs Private)	-	1.38 (1.33, 1.44)	1.37 (1.32, 1.43)
Smoking		0.00 (0.00, 0.00)	0.00 (0.00, 0.00)
Dental procedures in the last 12 months			
Cleaning		0.94 (0.90, 0.99)	0.96 (0.91, 1.01)
Fluoride		1.71 (1.64, 1.79)	1.72 (1.64, 1.80)
Sealant		0.93 (0.89, 0.98)	0.93 (0.89, 0.98)
Restorative		2.09 (2.01, 2.18)	2.09 (2.01, 2.18)
Extraction		1.06 (0.99, 1.13)	1.06 (0.99, 1.13)
Zip code-level			
% Less than HS (in 10% increase)			1.06 (1.02, 1.09)
ADA index (in 10 percentile increase)			1.04 (1.03, 1.06)

Aged 6-10 years

	Model A	Model B	Model C
	HR (95% CI)	HR (95% CI)	HR (95% CI)
Race/ethnicity			
Latino	1.12 (1.07, 1.18)	0.98 (0.93, 1.03)	0.95 (0.90, 1.00)
Black	1.09 (1.01, 1.20)	1.01 (0.92, 1.11)	1.00 (0.90, 1.10)
Other	1.01 (0.93, 1.10)	0.94 (0.88, 1.02)	0.95 (0.88, 1.02)
Age at visit	0.33 (0.30, 0.35)	0.36 (0.33, 0.38)	0.36 (0.33, 0.38)
Age at visit squared	1.04 (1.03, 1.04)	1.03 (1.03, 1.04)	1.03 (1.03, 1.04)
Sex (vs Male)	0.97 (0.93, 1.02)	0.97 (0.94, 1.01)	0.97 (0.94, 1.01)
Medical conditions			
Growth/Development	1.21 (1.10, 1.34)	1.16 (1.06, 1.27)	0.92 (0.67, 1.27)
Musculoskeletal/Connective tissue disorder	0.93 (0.66, 1.32)	0.91 (0.67, 1.26)	1.01 (0.91, 1.11)
Neurologic/Nerve problem	1.06 (0.96, 1.19)	1.01 (0.92, 1.12)	1.16 (1.06, 1.27)

© 2023 Choi SE et al. *JAMA Network Open.*

Insurance type (vs Private)	-	1.24 (1.19, 1.29)	1.23 (1.18, 1.28)
Smoking		0.63 (0.25, 1.60)	0.67 (0.26, 1.70)
Dental procedures in the last 12 months			
Cleaning		0.53 (0.50, 0.57)	0.54 (0.50, 0.57)
Fluoride		1.72 (1.64, 1.81)	1.71 (1.63, 1.80)
Sealant		0.95 (0.92, 0.99)	0.96 (0.92, 0.99)
Restorative		1.85 (1.78, 1.92)	1.84 (1.77, 1.91)
Extraction		1.22 (1.16, 1.28)	1.21 (1.15, 1.28)
Zip code-level			
% Less than HS (in 10% increase)			1.07 (1.04, 1.11)
ADA index (in 10 percentile increase)			1.02 (1.01, 1.03)

Aged 11-18 Years

	Model A	Model B	Model C
	HR (95% CI)	HR (95% CI)	HR (95% CI)
Race/ethnicity			
Hispanic	0.88 (0.83, 0.93)	0.82 (0.78, 0.85)	0.80 (0.77, 0.84)
Black	1.17 (1.06, 1.29)	1.02 (0.94, 1.12)	1.04 (0.95, 1.13)
Other	0.86 (0.79, 0.93)	0.85 (0.80, 0.91)	0.87 (0.81, 0.94)
Age at visit	1.19 (1.10, 1.28)	1.15 (1.07, 1.24)	1.15 (1.07, 1.23)
Age at visit squared	0.99 (0.99, 1.00)	0.99 (0.99, 1.00)	0.99 (0.99, 1.00)
Sex (vs Male)	0.98 (0.94, 1.02)	0.98 (0.95, 1.02)	0.98 (0.95, 1.01)
Medical conditions			
Growth/Development	1.24 (1.13, 1.35)	1.10 (1.02, 1.19)	1.25 (1.06, 1.47)
Musculoskeletal/ Connective tissue disorder	1.35 (1.12, 1.63)	1.26 (1.08, 1.49)	1.05 (0.98, 1.11)
Neurologic/Nerve problem	1.15 (1.07, 1.24)	1.05 (0.98, 1.12)	1.09 (1.02, 1.18)
Insurance type (vs Private)	-	1.35 (1.30, 1.40)	1.35 (1.30, 1.40)
Smoking		1.43 (1.32, 1.55)	1.42 (1.30, 1.54)
Dental procedures in the last 12 months			
Cleaning		0.59 (0.56, 0.61)	0.59 (0.56, 0.62)
Fluoride		1.95 (1.88, 2.03)	1.94 (1.86, 2.01)
Sealant		0.81 (0.78, 0.85)	0.81 (0.78, 0.84)
Restorative		2.31 (2.23, 2.39)	2.29 (2.21, 2.38)
Extraction		1.15 (1.07, 1.23)	1.14 (1.06, 1.22)
Zip code-level			
% Less than HS (in 10% increase)			1.01 (0.98, 1.04)
ADA index (in 10 percentile increase)			1.05 (1.04, 1.06)

Total population

	Model A	Model B	Model C
	HR (95% CI)	HR (95% CI)	HR (95% CI)
Race/ethnicity			

© 2023 Choi SE et al. *JAMA Network Open.*

Latino	1.14 (1.11, 1.18)	0.96 (0.93, 0.98)	0.94 (0.92, 0.97)
Black	1.18 (1.12, 1.25)	1.01 (0.96, 1.06)	1.01 (0.97, 1.06)
Other	1.09 (1.04, 1.14)	0.99 (0.95, 1.03)	1.00 (0.96, 1.04)
Age at visit	0.87 (0.86, 0.87)	0.87 (0.86, 0.88)	0.87 (0.86, 0.87)
Age at visit squared	1.00 (1.00, 1.00)	1.00 (1.00, 1.00)	1.00 (1.00, 1.00)
Sex (vs Male)	0.99 (0.97, 1.01)	0.99 (0.97, 1.01)	0.99 (0.97, 1.01)
Medical conditions			
Growth/Development	1.19 (1.13, 1.26)	1.11 (1.06, 1.17)	1.15 (1.01, 1.32)
Musculoskeletal/ Connective tissue disorder	1.21 (1.04, 1.42)	1.16 (1.01, 1.33)	1.04 (0.99, 1.09)
Neurologic/Nerve problem	1.13 (1.07, 1.19)	1.04 (1.00, 1.10)	1.11 (1.05, 1.16)
Insurance type (vs Private)	-	1.33 (1.30, 1.36)	1.32 (1.29, 1.35)
Smoking		1.37 (1.26, 1.48)	1.36 (1.25, 1.47)
Dental procedures in the last 12 months			
Cleaning		0.74 (0.72, 0.77)	0.75 (0.73, 0.77)
Fluoride		1.81 (1.76, 1.85)	1.80 (1.76, 1.85)
Sealant		0.88 (0.86, 0.90)	0.88 (0.86, 0.90)
Restorative		2.23 (2.18, 2.28)	2.22 (2.17, 2.27)
Extraction		1.13 (1.09, 1.17)	1.12 (1.09, 1.16)
Zip code-level			
% Less than HS (in 10% increase)			1.04 (1.02, 1.06)
ADA index (in 10 percentile increase)			1.04 (1.03, 1.05)

© 2023 Choi SE et al. *JAMA Network Open.*

eTable 3. Mediation Analysis Results: Time to First Tooth Decay Event

	Black vs White		Hispanic vs White		Other vs White	
	HR (95% CI)	RE % (95% CI)	HR (95% CI)	RE % (95% CI)	HR (95% CI)	RE % (95% CI)
**Overall population**						
Total effect	1.25 (1.21, 1.29)		1.14 (1.163, 1.107)		1.06 (1.03, 1.09)	
Total direct effect	1.02 (0.99, 1.05)	9.6 (-4.8, 20.8)	0.94 (0.964, 0.916)	-51.4 (-88.4, -25.2)	1.01 (0.98, 1.04)	5.9 (-60.8, 43.6)
Total indirect effect	1.22 (1.20, 1.23)	90.4 (79.2, 104.8)	1.21 (1.201, 1.217)	151.4 (125.2, 188.4)	1.05 (1.05, 1.06)	94.1 (56.4, 160.8)
Insurance	1.12 (1.11, 1.14)	54.5 (46.8, 63.9)	1.09 (1.083, 1.099)	69.6 (56.9, 85.1)	1.04 (1.04, 1.05)	75.1 (45.1, 130.1)
Fluoride application	1.04 (1.03, 1.05)	19.1 (15.5, 22.8)	1.04 (1.041, 1.046)	34.6 (28.8, 43.7)	1.03 (1.02, 1.03)	46.3 (30.3, 81.8)
Restorative	1.03 (1.02, 1.04)	15.4 (11.5, 18.6)	1.03 (1.029, 1.036)	25.9 (21.7, 31.3)	1.01 (1.00, 1.01)	14.4 (6.6, 26.2)
Cleaning	1.01 (1.00, 1.01)	2.0 (0.9, 3.1)	1.00 (1.00, 1.01)	2.2 (1.3, 3.1)	0.99 (0.99, 0.99)	-10.5 (-20.4, -5.6)
Sealant	1.01 (1.00, 1.01)	2.1 (1.3, 3.0)	NA	NA	0.99 (0.99, 1.000)	-1.5 (-3.6, 0.4)
Extraction	NA	NA	1.00 (1.00, 1.00)	0.8 (0.3, 1.4)	1.00 (1.00, 1.01)	3.7 (2.0, 8.2)
Smoking status	0.99 (0.997, 0.998)	-1.1 (-1.5, -0.7)	0.99 (0.99, 0.10)	-2.5 (-3.4, -1.8)	0.99 (0.99, 0.99)	-5.4 (-10.3, -3.3)
% Less than high school	1.01 (1.003, 1.009)	2.7 (1.7, 3.9)	1.01 (1.01, 1.02)	9.6 (5.3, 14.0)	1.00 (1.00, 1.01)	2.0 (0.7, 3.5)
Area Deprivation Index	0.99 (0.988, 0.993)	-4.3 (-5.9, -2.9)	1.01 (1.01, 1.02)	11.3 (8.9, 13.8)	0.98 (0.98, 0.99)	-29.9 (-51.1, -18.4)

	Age 0-5 Years					
	Black vs White		Hispanic vs White		Other vs White	
	HR (95% CI)	RE % (95% CI)	HR (95% CI)	RE % (95% CI)	HR (95% CI)	RE % (95% CI)
Total effect	1.23 (1.16, 1.31)		1.43 (1.39, 1.48)		1.34 (1.27, 1.40)	
Total direct effect	0.97 (0.91, 1.03)	-16.9 (-67.2, 11.1)	1.07 (1.04, 1.10)	18.6 (11.2, 24.6)	1.14 (1.07, 1.18)	43.8 (30.0, 50.8)
Total indirect effect	2.86 (2.24, 3.82)	116.9 (88.9, 167.2)	1.34 (1.32, 1.36)	81.4 (75.4, 88.8)	1.18 (1.16, 1.19)	56.2 (49.2, 70.0)
Insurance	1.96 (1.68, 2.33)	78.9 (59.0, 114.1)	1.13 (1.12, 1.15)	34.5 (30.1, 39.7)	1.07 (1.06, 1.08)	23.4 (19.8, 30.2)
Fluoride application	1.04 (1.03, 1.05)	18.5 (14.1, 26.9)	1.06 (1.05, 1.07)	16.8 (15.0, 18.6)	1.06 (1.05, 1.07)	19.6 (16.6, 24.5)
Restorative	1.03 (1.02, 1.05)	15.7 (9.1, 23.5)	1.08 (1.07, 1.09)	21.7 (19.5, 24.0)	1.05 (1.04, 1.06)	16.8 (14.0, 21.1)
Cleaning	NA	NA	NA	NA	1.00 (1.00, 1.00)	0.6 (0.2, 1.2)
Sealant	1.00	0.7	1.00	-0.5	NA	NA

© 2023 Choi SE et al. *JAMA Network Open.*

	(1.00, 1.00)	(0.2, 1.5)	(1.00, 1.00)	(-0.9, -0.2)		
Extraction	NA	NA	NA	NA	1.01 (1.00, 1.01)	3.3 (2.1, 4.7)
Smoking status	NA	NA	NA	NA	NA	NA
% Less than high school	1.06 (1.04, 1.09)	7.0 (4.3, 10.6)	1.02 (1.01, 1.03)	5.3 (3.3, 7.6)	1.01 (1.00, 1.01)	2.0 (1.3, 2.9)
Area Deprivation Index	0.96 (0.95, 0.97)	-3.8 (-5.6, -2.6)	1.01 (1.01, 1.02)	3.6 (2.8, 4.4)	0.98 (0.98, 0.99)	-6.2 (-8.2, -4.8)

	**Age 6-10 Years**				**Age 11-18 Years**	
	**Black vs White**		**Hispanic vs White**		**Black vs White**	
	HR (95% CI)	RE % (95% CI)	HR (95% CI)	RE % (95% CI)	HR (95% CI)	RE % (95% CI)
Total effect	1.18 (1.10, 1.30)		1.17 (1.13, 1.22)		1.21 (1.13, 1.28)	
Total direct effect	1.01 (0.94, 1.11)	-4.3 (-61.2, 41.4)	0.97 (0.92, 1.01)	-24.7 (-61.2, 5.1)	1.03 (0.96, 1.10)	12.5 (-28.0, 38.7)
Total indirect effect	1.18 (1.15, 1.20)	104.3 (58.6, 161.2)	1.21 (1.19, 1.24)	124.7 (94.9, 161.2)	1.17 (1.15, 1.20)	87.5 (61.3, 128.0)
Insurance	1.11 (1.10, 1.13)	67.4 (38.1, 112.2)	1.09 (1.07, 1.10)	52.7 (39.0, 73.5)	1.10 (1.09, 1.11)	53.4 (40.6, 82.7)
Fluoride application	1.04 (1.03, 1.05)	24.6 (14.5, 40.0)	1.05 (1.04, 1.05)	29.3 (22.1, 37.7)	1.03 (1.02, 1.04)	14.1 (7.1, 21.5)
Restorative	1.02 (1.01, 1.03)	12.8 (5.4, 22.2)	1.03 (1.03, 1.04)	22.0 (15.6, 29.0)	1.04 (1.03, 1.05)	20.7 (13.4, 30.3)
Cleaning	1.00 (1.00, 1.00)	0.0 (0.0, 0.0)	1.00 (1.00, 1.00)	0.0 (0.0, 0.0)	1.01 (1.00, 1.01)	3.3 (0.7, 6.7)
Sealant	1.00 (1.00, 1.00)	-1.2 (-2.5, -0.5)	1.00 (1.00, 1.00)	-0.7 (-1.2, -0.3)	1.01 (1.01, 1.01)	5.7 (3.7, 8.5)
Extraction	1.00 (1.00, 1.00)	0.0 (0.0, 0.0)	1.00 (1.00, 1.00)	1.9 (1.2, 2.7)	1.00 (1.00, 1.00)	-1.2 (-2.3, -0.5)
Smoking status	1.00 (1.00, 1.00)	0.0 (0.0, 0.0)	1.00 (1.00, 1.00)	0.0 (0.0, 0.0)	1.00 (1.00, 1.00)	-1.5 (-2.7, -0.7)
% Less than high school	1.01 (1.00, 1.01)	4.5 (2.4, 9.3)	1.02 (1.01, 1.03)	13.7 (7.6, 18.7)	1.00 (1.00, 1.00)	0.0 (0.0, 0.0)
Area Deprivation Index	0.99 (0.99, 1.00)	-3.7 (-8.6, -0.1)	1.01 (1.01, 1.01)	5.6 (2.9, 9.0)	0.99 (0.98, 0.99)	-7.1 (-11.0, -4.2)

* The relative effects were calculated as the percentage of the total effect associated with corresponding indirect or direct effects.
NA: Predictors selected as mediators (Supplemental Text S3) were included as covariates, not as mediators. Dental procedures (fluoride, restorative, cleaning, sealant, and extraction) were performed prior to the diagnosis of tooth decay.
HR = Hazard Ratio; RE = Relative effects

© 2023 Choi SE et al. *JAMA Network Open.*

eFigure 1. Cumulative Incidence and Cumulative Hazard of Tooth Decay by Race and Ethnicity Among Overall Population

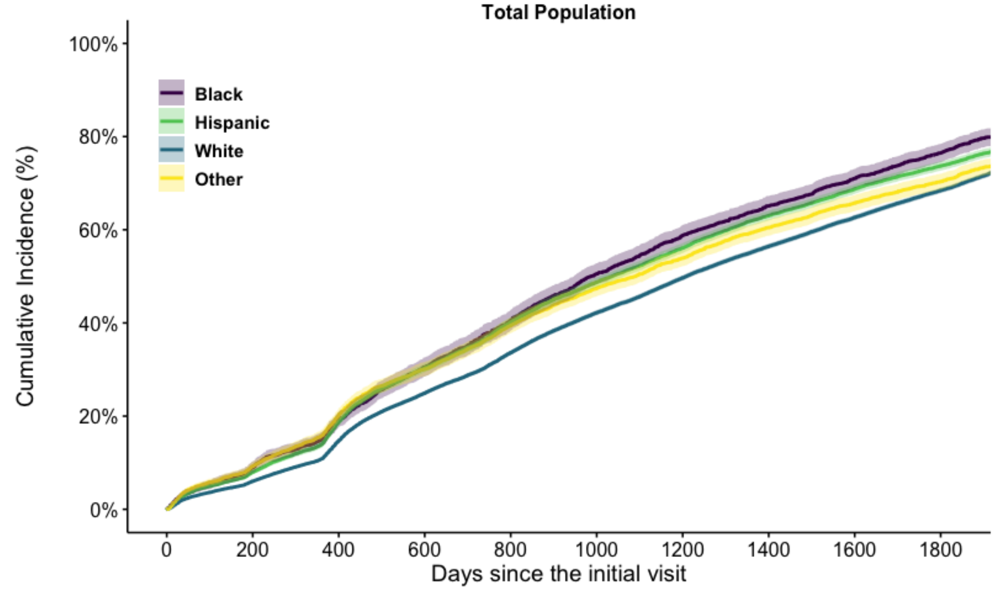

Number at risk

	0	200	400	600	800	1000	1200	1400	1600	1800
Black	2654	2420	2183	1932	1678	1424	1202	988	795	579
Hispanic	11213	10101	9256	8224	7185	6160	5169	4196	3390	2471
White	42815	39036	35592	31335	27292	23119	19355	15941	12617	9334
Other	4401	3952	3591	3207	2850	2497	2113	1732	1417	1098

Days since the initial visit

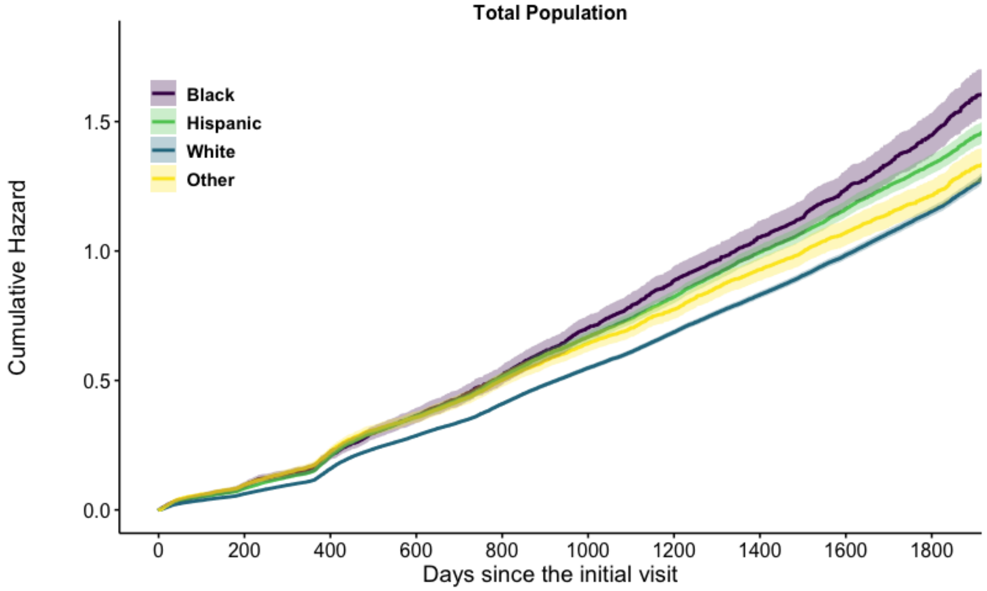

Number at risk

	0	200	400	600	800	1000	1200	1400	1600	1800
Black	2654	2420	2183	1932	1678	1424	1202	988	795	579
Hispanic	11213	10101	9256	8224	7185	6160	5169	4196	3390	2471
White	42815	39036	35592	31335	27292	23119	19355	15941	12617	9334
Other	4401	3952	3591	3207	2850	2497	2113	1732	1417	1098

Days since the initial visit

© 2023 Choi SE et al. *JAMA Network Open.*

eFigure 2. Cumulative Hazard of Tooth Decay by Race and Ethnicity and Age Groups

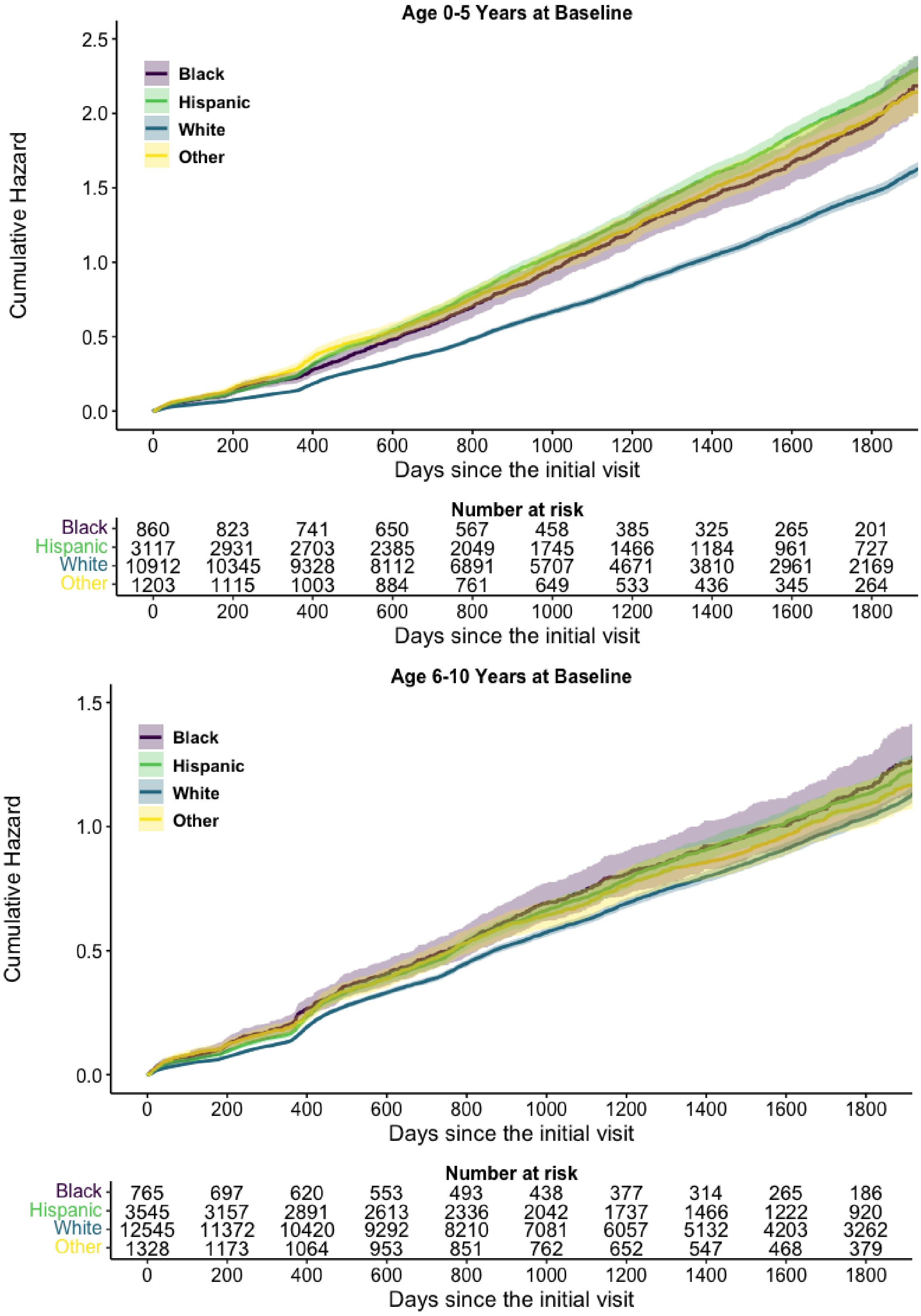

© 2023 Choi SE et al. *JAMA Network Open.*

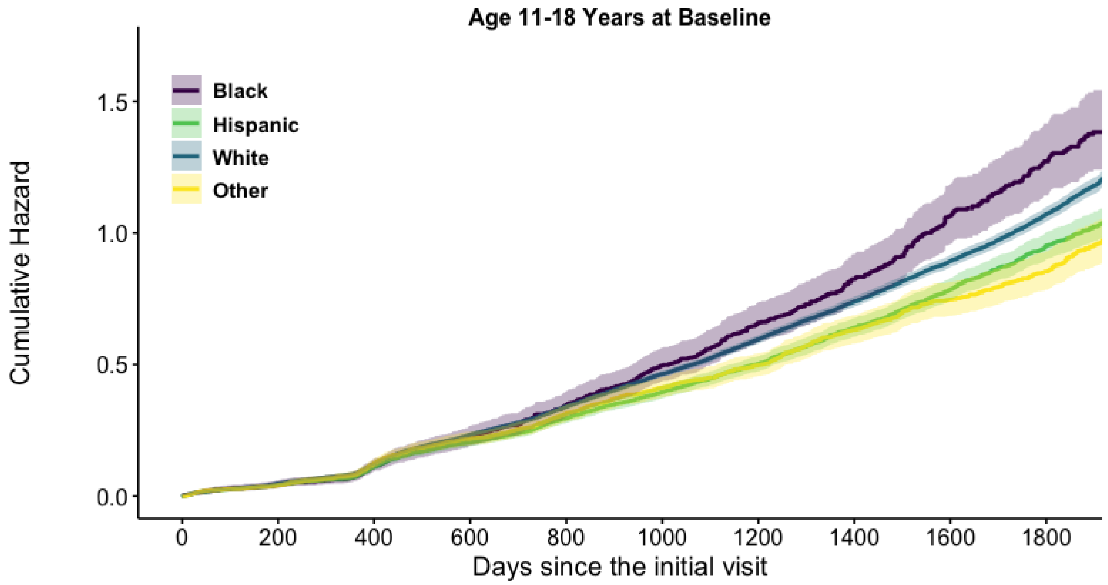

Number at risk

	0	200	400	600	800	1000	1200	1400	1600	1800
Black	1029	900	822	729	618	528	440	349	265	192
Hispanic	4551	4013	3662	3226	2800	2373	1966	1546	1207	824
White	19358	17319	15844	13931	12191	10331	8627	6999	5453	3903
Other	1870	1664	1524	1370	1238	1086	928	749	604	455

Days since the initial visit

Note: Cumulative hazard indicates the number of tooth decay events. "Other" racial/ethnic group included Asians, American Indians, and Hawaiian/Pacific Islanders. Shaded ribbons represent 95% confidence intervals

© 2023 Choi SE et al. *JAMA Network Open.*

eFigure 3. Validity of the Time-to-Event Model

Overall population

Estimated vs. observed 5-year risk of caries<br><br>AUC=0.71	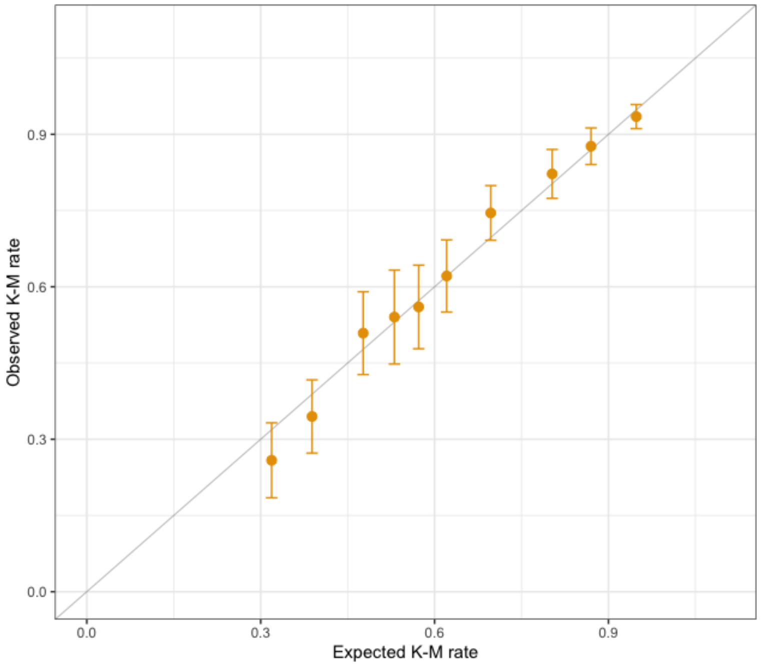
Estimated 5-year risk of developing caries vs. caries risk assessment at visit	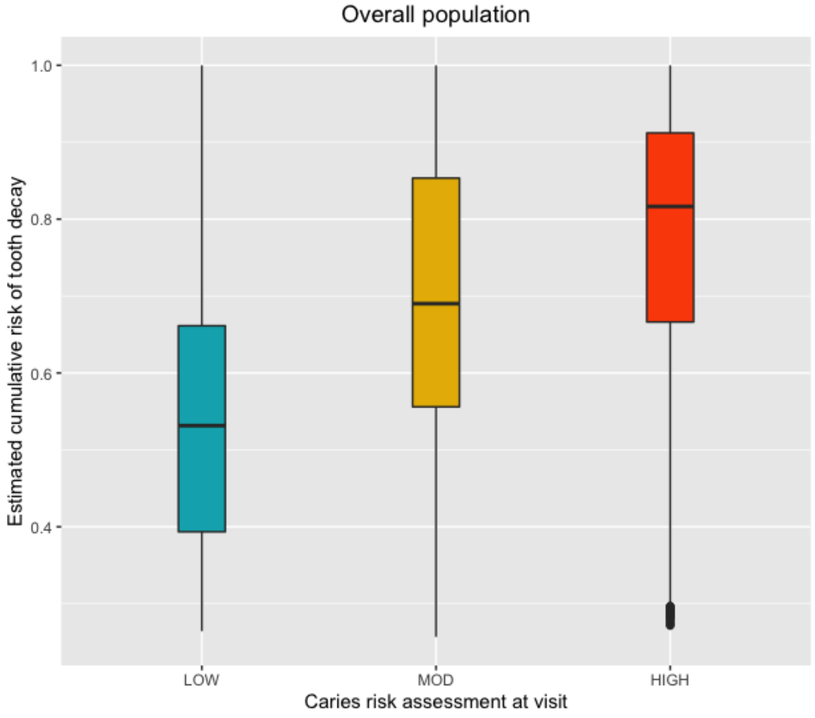<br>Caries risk assessment estimated.mean estimated.sd<br>LOW 0.55 0.18<br>MOD 0.70 0.17<br>HIGH 0.78 0.15

© 2023 Choi SE et al. *JAMA Network Open.*

Aged 0-5 Years

Estimated vs. observed 5-year risk of caries

AUC=0.685

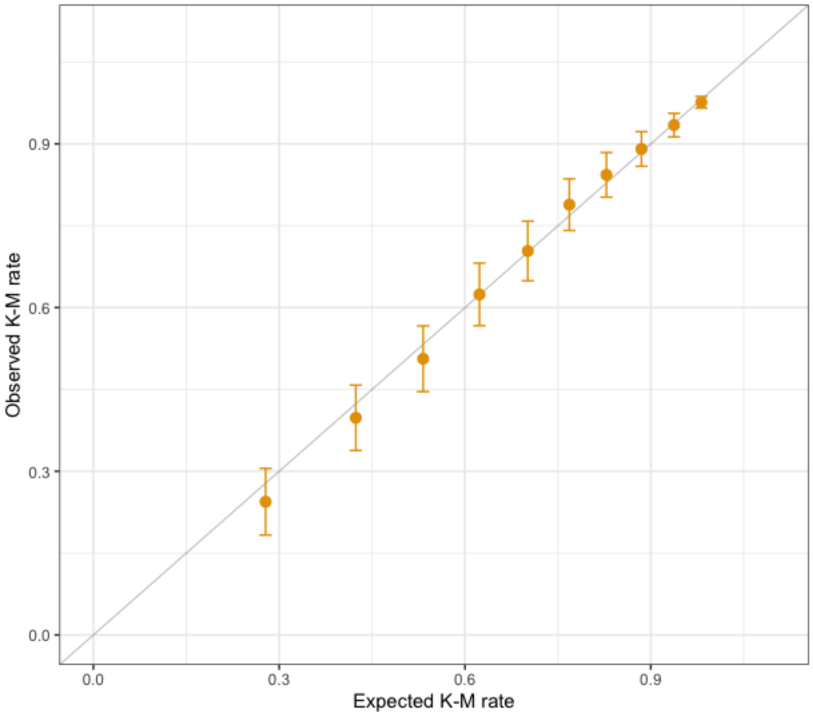

Estimated 5-year risk of developing caries vs. caries risk assessment at visit

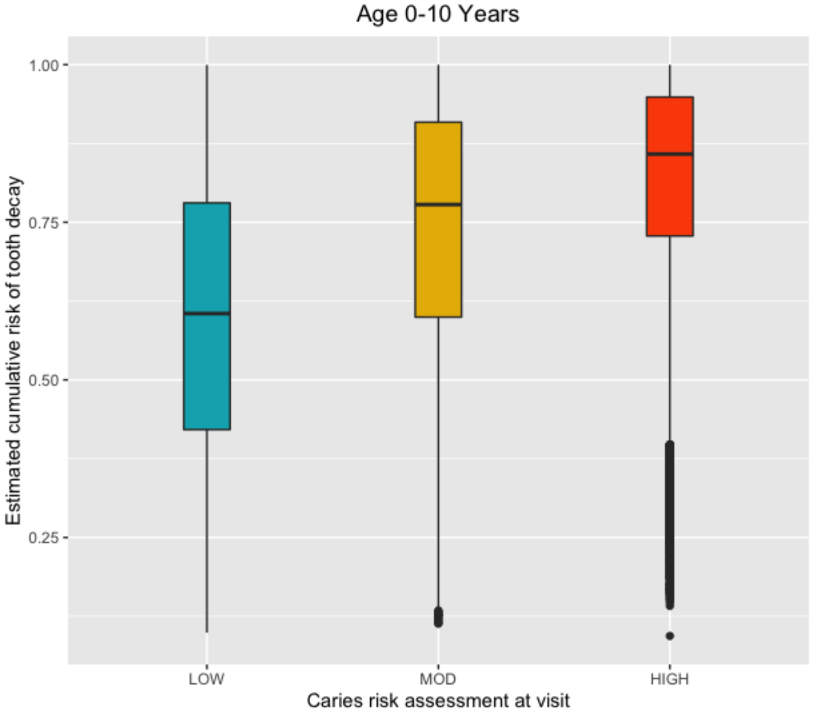

Caries risk assessment	estimated.mean	estimated.sd
LOW	0.59	0.22
MOD	0.73	0.20
HIGH	0.82	0.16

© 2023 Choi SE et al. *JAMA Network Open.*

Age 6-10 years

Estimated vs. observed 5-year risk of caries

AUC=0.70

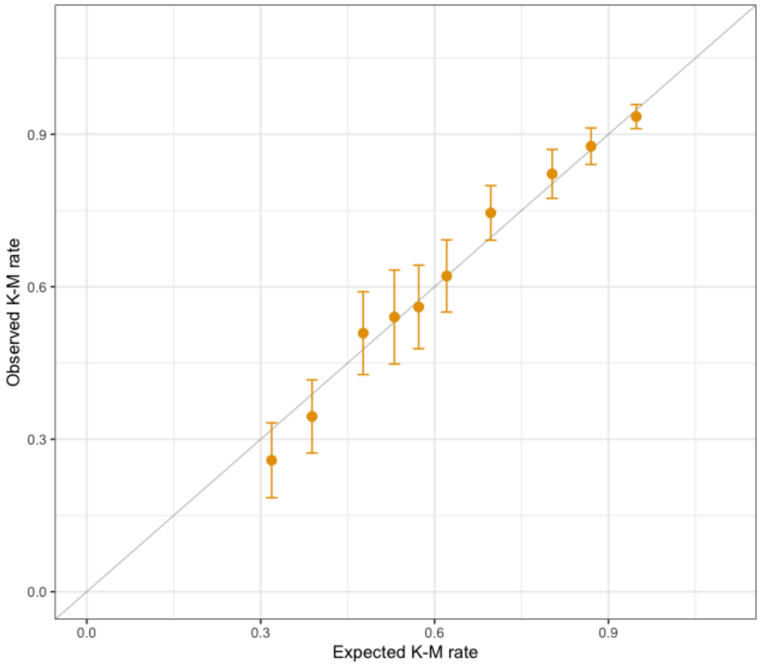

Estimated 5-year risk of developing caries vs. caries risk assessment at visit

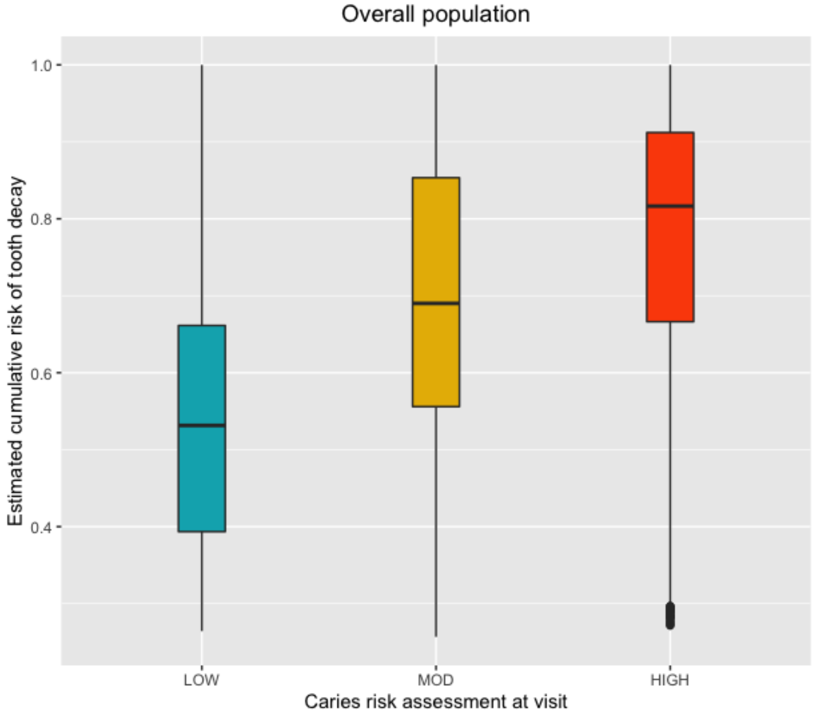

Caries risk assessment	estimated.mean	estimated.sd
LOW	0.54	0.17
MOD	0.71	0.17
HIGH	0.78	0.16

© 2023 Choi SE et al. *JAMA Network Open.*

Aged 11-18 Years

Estimated vs. observed 5-year risk of caries

AUC=0.655

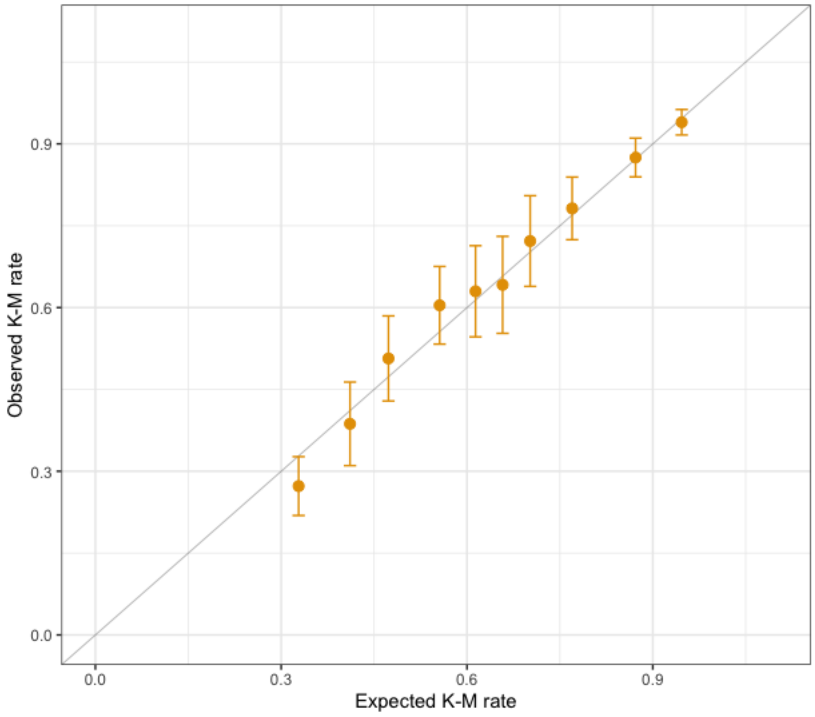

Estimated 5-year risk of developing caries vs. caries risk assessment at visit

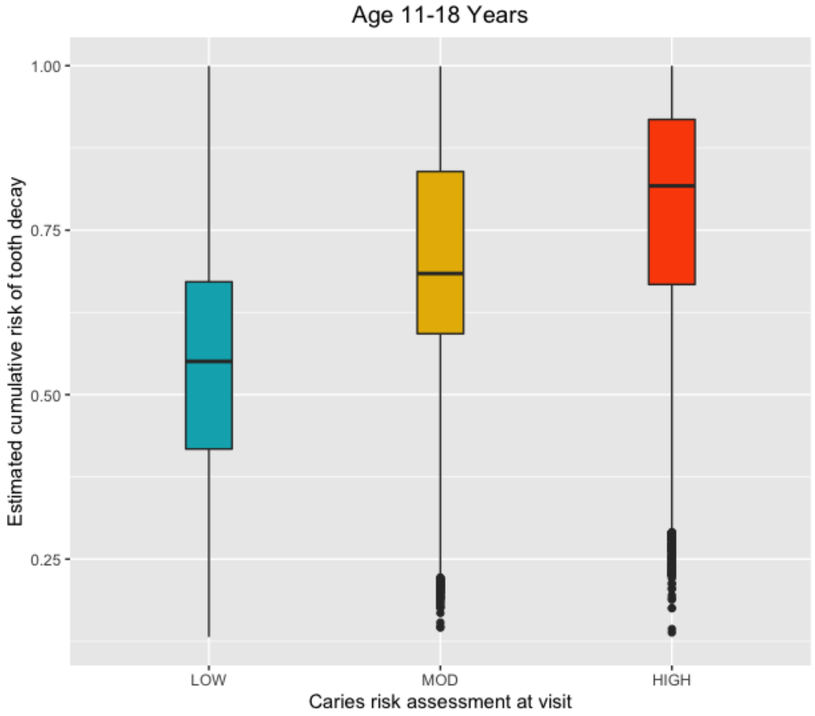

Caries risk assessment	estimated.mean	estimated.sd
LOW	0.55	0.16
MOD	0.69	0.16
HIGH	0.78	0.15

© 2023 Choi SE et al. *JAMA Network Open.*

References (Supplemental Appendix)

1. Barnard J, Schenker N, Rubin DB. Multiple Imputation In: Smelser NJS, Wright JD, eds. *International Encyclopedia of the Social & Behavioral Sciences.* 1st ed.: Elsevier; 2015.
2. White IR, Royston P, Wood AM. Multiple imputation using chained equations: Issues and guidance for practice. *Stat Med.* 2011;30(4):377-399.
3. Rubin D. Inference and missing data. *Biometrika.* 1976;63(3):581-592.
4. Simon N, Friedman J, Hastie T, Tibshirani R. Regularization Paths for Cox's Proportional Hazards Model via Coordinate Descent. *J Stat Softw.* 2011;39(5):1-13.
5. Tibshirani R, Bien J, Friedman J, et al. Strong rules for discarding predictors in lasso-type problems. *J R Stat Soc Series B Stat Methodol.* 2012;74(2):245-266.
6. Lasso and Elastic-Net Regularized Generalized Linear Models. https://cran.r-project.org/web/packages/glmnet/glmnet.pdf. Published 2022. Accessed May 7, 2022.
7. MacKinnon DP, Fairchild AJ, Fritz MS. Mediation analysis. *Annu Rev Psychol.* 2007;58:593-614.
8. Holland P. Statistics and Causal Inference. *J Am Stat Assoc.* 1986;81(396):945-960.
9. Yu Q, Li B, Scribner RA. Hierarchical additive modeling of nonlinear association with spatial correlations--an application to relate alcohol outlet density and neighborhood assault rates. *Stat Med.* 2009;28(14):1896-1912.
10. Yu Q, Li B. mma: An R Package for Mediation Analysis with Multiple Mediators. *Journal of Open Research Software.* 2017;5(1):11.

© 2023 Choi SE et al. *JAMA Network Open.*
