# Supplementary material for: Analysis of Race and Ethnicity, Socioeconomic Factors, and Tooth Decay Among US Children
Source: JAMA Netw Open. 2023 Jun 15;6(6):e2318425. doi: 10.1001/jamanetworkopen.2023.18425 (PMC10273024; doi:10.1001/jamanetworkopen.2023.18425)
Supplement: Supplement 2. — Data Sharing Statement [file jamanetwopen-e2318425-s002.pdf]

Data Sharing Statement

Choi. Analysis of Race and Ethnicity, Socioeconomic Factors, and Tooth Decay Among US Children. *JAMA Netw Open*. Published June 15, 2023.
doi:10.1001/jamanetworkopen.2023.18425

Data
Data available: No

Additional Information
Explanation for why data not available: The data that support the findings of this study were accessed under a data use agreement and are not publicly available.
